# Development and applications of a CRISPR activation system for facile genetic overexpression in *Candida albicans*

**DOI:** 10.1093/g3journal/jkac301

**Published:** 2022-12-01

**Authors:** Nicholas C Gervais, Alyssa A La Bella, Lauren F Wensing, Jehoshua Sharma, Victoria Acquaviva, Madison Best, Ricardo Omar Cadena López, Meea Fogal, Deeva Uthayakumar, Alejandro Chavez, Felipe Santiago-Tirado, Ana L Flores-Mireles, Rebecca S Shapiro

**Affiliations:** Department of Molecular and Cellular Biology, University of Guelph, Guelph, ON N1H 5N4, Canada; Department of Biological Sciences, University of Notre Dame, Notre Dame, IN 46556, USA; Department of Molecular and Cellular Biology, University of Guelph, Guelph, ON N1H 5N4, Canada; Department of Molecular and Cellular Biology, University of Guelph, Guelph, ON N1H 5N4, Canada; Department of Molecular and Cellular Biology, University of Guelph, Guelph, ON N1H 5N4, Canada; Department of Molecular and Cellular Biology, University of Guelph, Guelph, ON N1H 5N4, Canada; Department of Molecular and Cellular Biology, University of Guelph, Guelph, ON N1H 5N4, Canada; Department of Molecular and Cellular Biology, University of Guelph, Guelph, ON N1H 5N4, Canada; Department of Molecular and Cellular Biology, University of Guelph, Guelph, ON N1H 5N4, Canada; Present address: Department of Immunology, University of Toronto, Toronto, ON, Canada; Department of Pathology and Cell Biology, Columbia University College of Physicians and Surgeons, New York, NY 10032, USA; Department of Biological Sciences, University of Notre Dame, Notre Dame, IN 46556, USA; Department of Biological Sciences, University of Notre Dame, Notre Dame, IN 46556, USA; Department of Molecular and Cellular Biology, University of Guelph, Guelph, ON N1H 5N4, Canada

**Keywords:** fungal genetics, CRISPR activation, *Candida albicans*, gene regulation, fungal pathogens

## Abstract

For the fungal pathogen *Candida albicans*, genetic overexpression readily occurs via a diversity of genomic alterations, such as aneuploidy and gain-of-function mutations, with important consequences for host adaptation, virulence, and evolution of antifungal drug resistance. Given the important role of overexpression on *C. albicans* biology, it is critical to develop and harness tools that enable the analysis of genes expressed at high levels in the fungal cell. Here, we describe the development, optimization, and application of a novel, single-plasmid-based CRISPR activation (CRISPRa) platform for targeted genetic overexpression in *C. albicans*, which employs a guide RNA to target an activator complex to the promoter region of a gene of interest, thus driving transcriptional expression of that gene. Using this system, we demonstrate the ability of CRISPRa to drive high levels of gene expression in *C. albicans*, and we assess optimal guide RNA targeting for robust and constitutive overexpression. We further demonstrate the specificity of the system via RNA sequencing. We highlight the application of CRISPR activation to overexpress genes involved in pathogenesis and drug susceptibility, and contribute toward the identification of novel phenotypes. Consequently, this tool will facilitate a broad range of applications for the study of *C. albicans* genetic overexpression.

## Introduction

Fungal pathogens are an increasingly important cause of human illness, with significant impacts on morbidity and mortality (Fisher *et al.* 2020). *Candida albicans* is an opportunistic fungal pathogen that inhabits the microbiome of most healthy adults as a commensal organism (Gow *et al.* 2011; Pérez 2019). However, it is also responsible for invasive infections, particularly among immunocompromised individuals, which are often fatal (Bongomin *et al.* 2017). Instances of *C. albicans* infections are becoming more prevalent globally, in part due to an increasing population of susceptible elderly and immunocompromised individuals (Bongomin *et al.* 2017). Illustratively, systemic *Candida* infections have been identified in up to 15% of patients hospitalized for COVID-19, where patients with invasive candidiasis spent longer in the hospital intensive care unit on average and had more severe symptoms than those COVID-19 patients without a *Candida* infection (Segrelles-Calvo *et al.* 2021). The economic cost of *Candida* infections is estimated to be ∼1.4 billion dollars yearly in the United States of America alone, hospitalizing >26,000 people (Benedict *et al.* 2018). With limited development of novel antifungal drugs (Perfect 2017) and increasing prevalence of antifungal drug-resistant strains and species (Sanguinetti *et al.* 2015; Arendrup and Patterson 2017; Geddes-McAlister and Shapiro 2018), *Candida* pathogens remain a serious threat to human health.

Given the importance of *C. albicans* and other fungal pathogens, it is imperative that tools are developed and applied to manipulate these organisms and dissect genetic functions. For *C. albicans*, along with countless other microbial species, CRISPR-based genetic manipulation tools have revolutionized the ability of scientists to modify and study gene function (Shapiro, Chavez, Collins 2018; Uthayakumar *et al.* 2021). Such CRISPR platforms typically rely on a CRISPR-associated endonuclease protein, often Cas9, targeted to a genomic region of interest via a single-guide RNA (sgRNA), resulting in targeted DNA double-strand breaks, and subsequent repair via nonhomologous end-joining or homology-directed repair via a donor DNA repair template (Adli 2018; Bock *et al.* 2022). Since the initial development of CRISPR tools for genetic editing in *C. albicans* (Vyas *et al.* 2015), numerous systems have been developed to modify this fungal genome with efficiency and specificity (Min *et al.* 2016, 2018; Huang and Mitchell 2017; Ng and Dean 2017; Nguyen *et al.* 2017; Shapiro, Chavez, Porter, *et al.* 2018; Vyas *et al.* 2018; Román, Prieto, *et al.* 2019; Morio *et al.* 2020; Uthayakumar *et al.* 2021), and to build and screen genetic mutant libraries (Shapiro, Chavez, Porter, *et al.* 2018; Rosiana *et al.* 2021). Together, these gene editing systems have played an important role in providing novel biological insight into this fungal pathogen.

In addition to these canonical CRISPR-based genetic modification systems that introduce mutations, insertions, or deletions into the genome, alternative CRISPR technologies have been developed that modulate the expression of *C. albicans* genes (Román, Coman, *et al.* 2019; Wensing *et al.* 2019). These CRISPR systems rely on the inactivation of the Cas9 endonuclease, to generate a nuclease-dead (dCas9) protein, which can be fused to constructs from a transcriptional repressor or activator that generate CRISPR interference (CRISPRi) or CRISPR activation (CRISPRa) systems, respectively (Dominguez *et al.* 2016; Wang *et al.* 2016). These dCas9 fusions, when targeted to the promoter region of a gene via the sgRNA, can repress or activate gene transcription from a gene of interest, and have been widely applied in diverse organisms (Peters *et al.* 2015; Kampmann 2018; Xu and Qi 2019; Schultenkämper *et al.* 2020; Todor *et al.* 2021; Bock *et al.* 2022). In *C. albicans*, these modified CRISPR systems have enabled the modulation and study of important fungal genes, including essential genes (Román, Coman, *et al.* 2019; Wensing *et al.* 2019; Wensing and Shapiro 2022).

While a majority of CRISPR systems and other genetic manipulation platforms focus on gene mutation, deletion, or downregulation, it is also critical to develop techniques that enable the upregulation or overexpression of genes. Indeed, genetic overexpression in *C. albicans* occurs readily via gene copy number amplification and aneuploidy, and these copy number amplification events are frequently observed in genetically diverse clinical isolates (Coste *et al.* 2006; Forche *et al.* 2018; Todd *et al.* 2019). The upregulation of gene expression plays a critical role in diverse aspects of fungal biology (Rai *et al.* 2021), including the evolution of antifungal drug resistance (Selmecki *et al.* 2008; Sanglard *et al.* 2009; Schubert *et al.* 2011; Flowers *et al.* 2012; Lohberger *et al.* 2014; Todd and Selmecki 2020), host colonization (Prieto *et al.* 2017; Znaidi *et al.* 2018), and virulence (Fu *et al.* 2008; Lohberger *et al.* 2014). Additionally, in other microbial systems, including bacteria, protozoa, and the model yeast *Saccharomyces cerevisiae*, genetic overexpression libraries have proved to be critical tools for identifying targets of drugs with uncharacterized mechanisms of action, as mutants overexpressing the drug target are typically more resistant to the drug in question (Li *et al.* 2004; Luesch *et al.* 2005; Smith *et al.* 2010; Begolo *et al.* 2014). Overexpression screens can also help dissect molecular pathways (Sopko *et al.* 2006; Sahni *et al.* 2010; Polvi *et al.* 2019). In *C. albicans*, several techniques exist for genetic overexpression (Rai *et al.* 2021), including promoter replacement strategies (Delgado *et al.* 2003; Park and Morschhäuser 2005; Eckert and Mühlschlegel 2009; Sahni *et al.* 2010), the generation of ORFeome collection strains (Chauvel *et al.* 2012; Legrand *et al.* 2018), and CRISPR-based methods (Román, Coman, *et al.* 2019). While these tools have made important contributions to understanding overexpression in *C. albicans*, there remains a need for simple and rapid methods to efficiently target fungal genes of interest for overexpression, and to do so in diverse strain backgrounds.

Here, we introduce a CRISPR-based tool for genetic overexpression in *C. albicans*, to bolster the existing functional genetic toolbox available to manipulate genes in this critical fungal pathogen. Our CRISPRa tool exploits a *C. albicans*-optimized tripartite activator complex fused to dCas9, which was previously developed for highly efficient transcriptional regulation in *S. cerevisiae* (Chavez *et al.* 2015). This CRISPRa system is distinct from other CRISPR-based gene activation systems for *C. albicans*, as it is an efficient single-plasmid system designed for rapid Golden Gate cloning and facile fungal strain generation, which can be readily applied to diverse strain backgrounds including clinical isolates. We test CRISPRa guide targeting principles based on predicted transcriptional start sites, confirm on-target guide efficiency, and demonstrate the ability of this CRISPRa system to drive high levels of expression of *C. albicans* genes. Using this optimized CRISPRa technique, we validate its ability to drive the overexpression of genes with established roles in antifungal drug susceptibility and biofilm formation. Together, this work introduces a novel CRISPRa system for genetic overexpression in *C. albicans*, with a wide range of future applications.

## Materials and methods

### Plasmid design and cloning

The plasmid backbone used in this study was the *C. albicans*-optimized CRISPR-dCas9 plasmid (pRS143, Addgene #122377) used in our previous study (Wensing *et al.* 2019), containing the *NEUT5L* integration site, sgRNA cloning site (*SNR52* promoter, SapI cloning locus, and sgRNA tail), and *dCAS9*. The dCas9-VP64-p65-Rta (VPR) fusion construct was generated via Gibson assembly. The VPR tripartite complex was codon-optimized for *C. albicans* expression and synthesized as gBlocks gene fragments from Integrated DNA Technologies (IDT). This gene fragment was cloned with Gibson assembly into the *dCAS9* plasmid backbone. We have made the CRISPRa (dCas9-VPR) plasmid available via Addgene (reference #182707). All plasmids are listed in Supplementary Table 1.

### sgRNA design and cloning

CRISPRa strain construction was broadly performed as described in detail (Wensing and Shapiro 2022), with some minor modifications. The sgRNA CRISPR RNAs (crRNA; 20 nucleotide sequence complementary to the target DNA genomic DNA) were designed based on efficiency and predicted specificity via the sgRNA design tool Eukaryotic Pathogen CRISPR gRNA Design Tool (EuPaGDT; http://grna.ctegd.uga.edu; Peng and Tarleton 2015). Generally, designing sgRNAs around −100 to −350 bp upstream of both the start codon (ATG) and transcriptional start site of the target gene led to most successful overexpression. In all cases, employing 4-6 sgRNAs in total (2-3 sgRNAs per site) led to significant overexpression in at least one of the corresponding strains. These sgRNA crRNA N20 sequences were cloned into the dCas9-VPR CRISPRa plasmid at the sgRNA cloning locus (SapI cloning site, flanked by *SNR52* promoter, and sgRNA tail) using Golden Gate cloning (Engler *et al.* 2008), as previously described (Halder *et al.* 2019; Wensing and Shapiro 2022). crRNA N20 sequences were obtained as two oligonucleotides from IDT in forward and reverse complement orientation, each containing a SapI cloning site, and were reconstituted to 100 µM in a nuclease-free duplex buffer from IDT. Equal volumes of the two complementary oligonucleotides were combined after being heated separately at 94°C, and then duplexed by heating to 94°C for 2 min and cooling to room temperature. The duplexed fragment was then cloned into the CRISPRa plasmid with the following Golden Gate cloning reaction: 10 µl miniprepped CRISPRa plasmid, 1 µl duplexed oligonucleotide, 2 µl 10X CutSmart buffer, 2 µl ATP, 1 µl SapI, 1 µl T4 DNA ligase, and 3 µl nuclease-free water. This mixture was incubated in a thermocycler under the following cycling conditions: (37°C, 2 min; 16°C, 5 min) for 99 cycles; 65°C, 15 min; 80°C, 15 min. After cycling, 1 µl of additional SapI was added to each reaction mixture, and the reaction was incubated at 37°C for 1 h. All sgRNAs are listed in Supplementary Table 1.

### Media and growth conditions

*Escherichia coli* DH5α cells were grown at 37°C in Lysogeny Broth (LB) and LB plates supplemented with 100 mg/ml ampicillin (AMP) and 250 mg/ml nourseothricin (NAT) for plasmid selection. *Candida albicans* cells were grown at 30°C or 37°C in yeast peptone dextrose (YPD) broth and YPD plates supplemented with 250 mg/ml NAT for plasmid selection.

### Bacterial transformation

High-efficiency 5-alpha competent *E. coli* (NEB) cells were thawed on ice. 1µl of Golden Gate cloning reagents was added to 50 µl of competent cells and incubated on ice for 30 min, heat shocked for 30 s at 42°C, and incubated on ice for an additional 5 min. About 950 µl of SOC outgrowth media was added to each cell culture and incubated at 30°C for 1.5 h at 250 RPM. Transformed cells were then plated on LB media containing AMP and NAT and grown at 30°C for 1 day.

### *Candida albicans* transformation

Plasmids were transformed into *C. albicans* via a chemical transformation strategy, as previously described (Halder *et al.* 2019; Wensing and Shapiro 2022). Briefly, miniprepped CRISPRa plasmids were linearized via a 1.5X restriction digest mix using the PacI enzyme. A transformation master mix was prepared as follows: 800 µl of 50% polyethylene glycol, 100 µl of 10X Tris-EDTA buffer solution, 100 µl of 1 M lithium acetate, 40 µl of 10 mg/ml salmon sperm DNA, and 20 µl of 1 M dithiothreitol. The transformation mix was added to *C. albicans* cells and linearized CRISPRa plasmid and left to incubate at 30°C for 1 h, then heat shocked at 42°C for 45 min. Cells were washed with fresh YPD, then grown in YPD for 4 h to allow for expression of the NAT resistance construct. Transformed cells were then plated on YPD media containing NAT, and grown at 30°C for 2 days. All *C. albicans* strains are listed in Supplementary Table 1.

### Growth curves

Overnight cultures of *C. albicans* grown in YPD were diluted to an OD_600_ of 0.05 in fresh YPD and added to a 96-well flat-bottomed plate with a total volume of 200 µl per well. Growth was measured at 30°C via optical density at 600 nm at 15 min intervals over the course of 24 h using an Infinite 200 PRO microplate reader (Tecan), and plates were shaken orbitally for 900 s at a 4-mm amplitude in between growth measurements.

### Minimum inhibitory concentration assays

Fluconazole minimum inhibitory concentration (MIC) assays were performed in 96-well flat-bottomed plates. A suspension of 40 μg/ml fluconazole was prepared in water, of which 100 μl was added to the first column of each plate containing 100 μl of culture, to obtain a total volume of 200 µl per well. The first column was serially diluted 2-fold across the plate in water. The gradient of fluconazole, therefore, ranged from 20 to 0 μg/ml. Overnight cultures of *C. albicans* grown in YPD were diluted to an OD_600_ of 0.1 in 2X RPMI-1640 with 40 g/l of added D-glucose and mixed into the plates in an equal volume such that the starting OD_600_ values of each strain were 0.05 and the growth media was diluted to RPMI-1640 with 20 g/l of added D-glucose. Plates were incubated at 37°C at 900 RPM, and absorbance values at 600 nm were read after 24 h using an Infinite 200 PRO microplate reader (Tecan). The amphotericin B MIC assays were also performed in 96-well flat-bottomed plates similar to the protocol described above with a few modifications. A starting concentration of 5 μg/ml of amphotericin B was used to create a gradient ranging from 2.5 to 0 μg/ml. Strains were instead grown in YPD media using a starting OD_600_ of 0.001. Plates were incubated at 37°C statically and absorbance values at 600 nm were read after 72 h using an Infinite 200 PRO microplate reader (Tecan).

### Biofilm assays

RPMI-based biofilm assays were performed as previously described (Razzaq *et al.* 2021), with minor modifications. Overnight cultures of *C. albicans* grown in YPD were diluted to an OD_600_ of 0.001 in 5 ml of RPMI-1640 with 20 g/l of supplemented D-glucose. 100µl of RPMI-1640 was mixed with 100 µl of each diluted overnight culture into a 96-well flat-bottomed plate (12 wells per strain). Plates were grown statically at 37°C for 24 h to allow biofilms to form. About 120 µl of planktonic cell supernatant was removed from each well and moved to a new 96-well flat-bottomed plate. Density of the planktonic cells was measured at 600 nm using an Infinite 200 PRO microplate reader (Tecan). Original plates from which planktonic cells were removed were washed by adding 200 µl 1X PBS to each well with an electronic multichannel pipette at a moderate dispense speed. 1X PBS was then discarded, and this wash step was repeated. Plates were then left to dry for 1–2 h. Once biofilms were dry, 90 µl of 1 mg/ml tetrazolium salt (XTT) (prepared in 1X PBS and centrifuged to remove sediment prior to use) and 10 µl of 0.32 mg/ml PMS (prepared in water) were added to each well. Plates were incubated statically at 30°C for 2 h to allow biofilms to reduce XTT, measured at 490 nm using an Infinite 200 PRO microplate reader (Tecan), and normalized to the growth of planktonic cells harvested previously.

For urine-based biofilm assays, biofilm formations were performed in 96-well flat-bottomed plates. Plates were coated with 150 µg/ml of fibrinogen and incubated overnight at 4°C. The *C. albicans* strains were cultured at 37°C with aeration in 5 ml of YPD broth. The inoculum was normalized to ∼1 × 10^6^ CFU/ml and then diluted (1:10) in human urine (female, pH ∼6.5). Urine was supplemented with 20 mg/ml of bovine serum albumin (BSA) for carbon and nitrogen sources mimicking the catheterized bladder environment. 200µl of the urine with inoculum was incubated in each well of the 96-well plate at 37°C for 48 h while static. Following the 48 h incubation, the cultures were removed and the wells were washed 3× with 200 µl 1X PBS to remove unbound fungi. Plates were incubated with 200 µl of 0.5% crystal violet for 15 min. Crystal violet was removed, and plates were washed with water and dried. 200µl of 33% acetic acid was added to the wells and 100 µl was transferred to a new 96-well plate. Absorbance was measured at 595 nm using a SpectraMax ABS Plus microplate reader (Molecular Devices).

### RNA extraction and real-time quantitative PCR

To detect differences in gene expression, overnight cultures of *C. albicans* grown in YPD were diluted to an OD_600_ of 0.05 in fresh YPD and grown to an OD_600_ of >0.2 at 30°C. *ALS3* CRISPRa strains were diluted to an OD_600_ of 0.05 in fresh RPMI and grown for 5 h at 37°C as well to measure overexpression in conditions that favor high levels of basal expression. Cultures were pelleted and frozen at −80°C before RNA was extracted. RNA extractions were performed using either a Presto Mini RNA Yeast kit from FroggaBio (cat. RBYD050) or an RNeasy Mini kit from Qiagen (cat. 74104). To assess RNA integrity, an RNA ScreenTape assay was performed on all samples using the TapeStation 4150 system following manufacturer's instructions (Agilent Technologies). Only samples with RIN values of >5.0 were used for reverse-transcription quantitative PCR (RT-qPCR). Synthesis of cDNA from 1,000 ng (10 µl) RNA was performed using a High Capacity cDNA Reverse-Transcription kit from Applied BioSystems (cat. 4368814). Briefly, 20 μl reaction mixtures were prepared with 2 μl 10X reverse-transcription buffer, 0.8 μl of 25X dNTPs at a concentration of 100 mM, 2 μl of random primers, 1 μl of MultiScribe Reverse Transcriptase at 50 IU/μl, 4.2 μl of nuclease-free water, and 10 μl of RNA (1 μg). Reverse-transcription run conditions were as follows: 10 min at 25°C, 120 min at 37°C, 5 min at 85°C. Real-time PCR assays were conducted using a QuantStudio 7 Pro Real-Time PCR system from Thermo Fisher Scientific Inc. 20 μl reaction mixtures containing 10 μl 2X SsoAdvanced Universal Inhibitor-Tolerant SYBR supermix (Bio-Rad, cat: 172-5017), 0.8 μl of PCR forward and reverse primer mix at 5 μM (final concentration of primer at 200 nM), 4.2 μl of water, and 5 μl of diluted cDNA. The run conditions were as follows: 3 min at 98°C polymerase activation step, followed by 40 cycles of a two-step qPCR (10 s of 98°C denaturation, 30 s of 60°C combined annealing/extension). Primers are listed in Supplementary Table 1. Expression profiling calculations were performed according to the comparative *C_T_* method (Schmittgen and Livak 2008). Briefly, expression values for the experimental gene of interest were compared with the housekeeping gene *ACT1* to obtain a Δ*C_T_* value within each strain. The Δ*C_T_* values in the experimental CRISPRa strains were then compared with the nontargeting CRISPRa control strain to obtain a ΔΔ*C_T_* value and finally a fold difference in expression of the experimental gene, and are listed in Supplementary Table 3.

### RNA sequencing

RNA preparation and sequencing (RNA-seq) were performed as previously described, with minor modifications (Razzaq *et al.* 2021). Strains were grown in YPD at 30°C overnight preceding RNA extraction. RNA extraction, sample QC, library preparations, and sequencing reactions were conducted at GENEWIZ, LLC./Azenta US, Inc. as follows: Total RNA was extracted using Qiagen RNeasy Plus Universal kit following manufacturer's instructions (Qiagen). RNA samples were quantified using Qubit 2.0 Fluorometer (Thermo Fisher Scientific) and RNA integrity was checked with 4200 TapeStation (Agilent Technologies). ERCC RNA Spike-In Mix kit (cat. 4456740) from Thermo Fisher Scientific was added to normalized total RNA prior to library preparation following the manufacturer's protocol. The RNA-seq library was prepared using the NEBNext Ultra II RNA Library Prep kit for Illumina using the manufacturer's instructions (New England Biolabs). Briefly, mRNAs were initially enriched with Oligod(T) beads. Enriched mRNAs were fragmented for 15 min at 94°C. First-strand and second-strand cDNAs were subsequently synthesized. cDNA fragments were end-repaired and adenylated at 3′ ends, and universal adapters were ligated to cDNA fragments, followed by index addition and library enrichment by PCR with limited cycles. The sequencing library was validated on the Agilent TapeStation (Agilent Technologies), and quantified by using Qubit 2.0 Fluorometer (Thermo Fisher Scientific) as well as by quantitative PCR (KAPA Biosystems). The sequencing libraries were multiplexed and clustered onto a flowcell. After clustering, the flowcell was loaded onto the Illumina HiSeq 4000 or equivalent instrument according to the manufacturer's instructions. The samples were sequenced using a 2 × 150 bp paired end configuration. Image analysis and base calling were conducted by the Illumina Control Software.

Raw sequence data (.bcl files) generated from the Illumina instrument was converted into fastq files and de-multiplexed using Illumina bcl2fastq 2.20 software. One mis-match was allowed for index sequence identification. Sequence reads were trimmed to remove possible adapter sequences and nucleotides with poor quality using Trimmomatic v.0.36, and trimmed reads were mapped to the candida_albicans reference genome available on ENSEMBL using the STAR aligner v.2.5.2b. The STAR aligner is a splice aligner that detects splice junctions and incorporates them to help align the entire read sequences. BAM files were generated as a result of this step. Unique gene hit counts were then calculated by using featureCounts from the Subread package v.1.5.2. The hit counts were summarized and reported using the gene_id feature in the annotation file. Only unique reads that fell within exon regions were counted. After extraction of gene hit counts, the gene hit counts table was used for downstream differential expression analysis. Using DESeq2, a comparison of gene expression between the control and experimental groups of samples was performed. The Wald test was then used to generate *P*-values and log2-fold changes. Genes with an adjusted *P*-value <0.05 and absolute log2-fold change >1 were called as differentially expressed genes for each comparison. The data have been deposited in NCBI's Gene Expression Omnibus (GEO) (Edgar *et al.* 2002) and are accessible through GEO Series accession number GSE201904 (https://www.ncbi.nlm.nih.gov/geo/query/acc.cgi?acc=GSE201904).

## Results

### Development of a single-plasmid CRISPRa tool in *C. albicans*

First, we designed a single-plasmid CRISPRa system for targeted gene activation, optimized for *C. albicans*. We exploited an integrating plasmid backbone, which we previously used for CRISPRi in *C. albicans*, containing a codon-optimized dCas9 and a Golden Gate assembly compatible sgRNA cloning site (Wensing *et al.* 2019). This plasmid can be linearized to integrate at the *C. albicans NEUT5L* locus (a large intergenic region whose disruption does not affect fungal fitness; Gerami-Nejad *et al.* 2013), and contains a dual SapI cloning site for facile and highly efficient Golden Gate cloning of the sgRNA targeting sequence. This plasmid also contains a dominant NAT resistance cassette, to enable strain generation in a diversity of fungal strains, including clinical isolates.

**Fig. 1.**
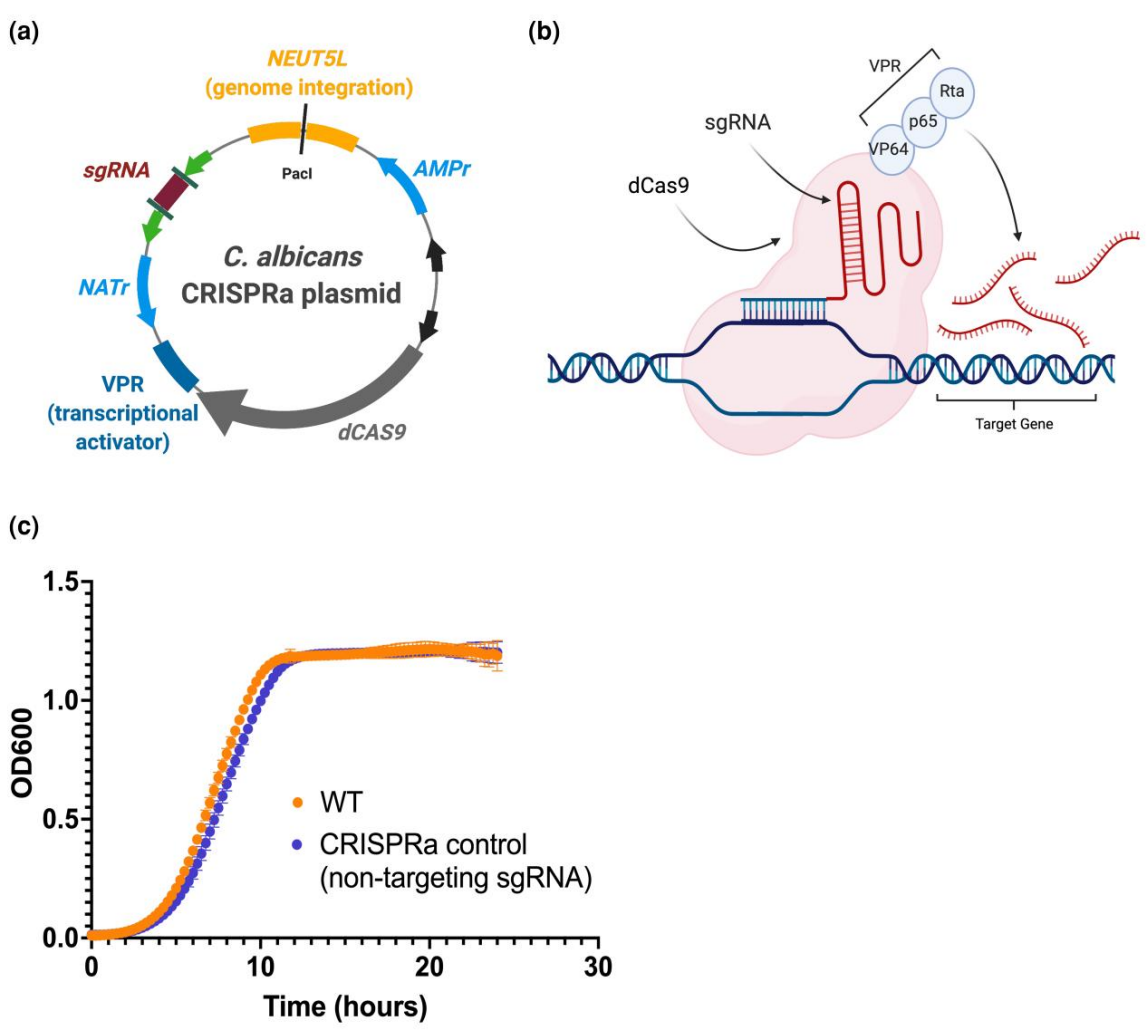
Design of a CRISPRa system for *Candida albicans*. a) The single-plasmid system contains a catalytically inactive (“dead”) dCas9 protein, paired with the VPR complex of activator domains to cause upregulation at a target locus. The sgRNA region can be subjected to Golden Gate cloning with a custom N20 sequence in order to target the dCas9-VPR complex to a desired region of DNA through complementary base-pairing. The PacI enzyme is used to linearize the plasmid at the *NEUT5L* locus, which allows the plasmid to be integrated into the cell's chromosome and remain in the cell following cell division. Panel created with BioRender.com. b) Once the sgRNA recognizes and binds to a target sequence of DNA, the dCas9-VPR complex will cause the recruitment of transcription initiation complex factors to the adjacent genetic material, thus increasing gene expression from that region. Panel created with BioRender.com. c) Wild-type *C. albicans* cells and cells containing a nontargeting CRISPRa plasmid were grown in YPD at 30°C to compare fitness under standard laboratory conditions. Growth was recorded via optical density at 600 nm at 15 min intervals over the course of 24 h. Data points show the mean and standard deviation at each time point (*n* = 32).

To generate a version of dCas9 that could lead to strong constitutive activation of genes of interest, we generated a plasmid with dCas9 fused to the tripartite activator complex, VPR (Fig. 1, a and b). VPR consists of 3 previously described transcriptional activators: (1) VP64, a viral transcriptional activator domain composed of 4 tandem copies of VP16 (Herpes simplex viral protein 16), which has been used extensively in CRISPRa systems (La Russa and Qi 2015); (2) p65, the NF-κB subunit responsible for the strong transcription activation in mammalian cells (Schmitz and Baeuerle 1991); and (3) Rta, the Epstein–Barr virus R transactivator, with strong transcriptional activation properties (Hardwick *et al.* 1992; Fig. 1, a and b). Together the VPR activator complex fused to dCas9 and targeted to the promoter region of genes has previously been demonstrated to generate robust gene activation in *S. cerevisiae* (Chavez *et al.* 2015). Exploiting the ability of dCas9 to bind and be targeted to genomic loci via sgRNAs enables us to design sgRNAs that will target dCas9 along with these transcriptional activators to any gene promoter of interest.

This plasmid was generated by homology-based cloning and validated via Sanger sequencing, and has been deposited to Addgene (Supplementary Table 1, Addgene plasmid #182707). To confirm if the expression of the dCas9-VPR construct had an impact on fungal fitness, we compared the growth of a wild-type *C. albicans* strain with a strain transformed with the dCas9-VPR CRISPRa plasmid (expressing a nontargeting sgRNA: a random sequence of nucleotides that have no predicted complementarity to any region in the *C. albicans* genome). We found no difference in growth between these strains (Fig. 1c), suggesting no significant impact of the CRISPRa system on *C. albicans* fitness.

### Validation and sgRNA design principles for CRISPRa overexpression in *C. albicans*

Next, we aimed to validate that the CRISPRa system could successfully overexpress genes in *C. albicans*, and sought to determine the optimal sgRNA design strategy to maximize genetic regulation. Our previous *C. albicans* CRISPRi system exploited sgRNAs ∼50–150 bp upstream of the start codon to functionally repress genes of interest (Wensing *et al.* 2019). However, CRISPRa systems are known to have different optimal sgRNA targeting regions (Jensen 2018; Sanson *et al.* 2018), and our previous design did not take into account the transcription start site (TSS), which is known to be an important predictor of regulatory efficiency for CRISPRi/a systems (Smith *et al.* 2016; Jensen 2018), but which had not been robustly characterized across all *C. albicans* genes at the time. More recently, the TSS for many *C. albicans* genes has been predicted with single-nucleotide resolution using nAnT-iCAGE technology, which specifically identifies the 5′ end of RNA fragments (Lu and Lin 2021). Thus, we sought to determine the optimal design for CRISPRa sgRNAs based on the start codon and the TSS. We identified 3 *C. albicans* genes with a predicted TSS that was particularly far upstream of the start codon compared with all other genes in the genome (801–952 bp upstream): *KSP1*, *ALS3*, and *FGR28*. We designed 6 sgRNAs for each gene, targeting upstream of the start codon (as previously designed for CRISPRi; Wensing *et al.* 2019), as well as upstream of the TSS (based on predicted TSS; Lu and Lin 2021).

**Fig. 2.**
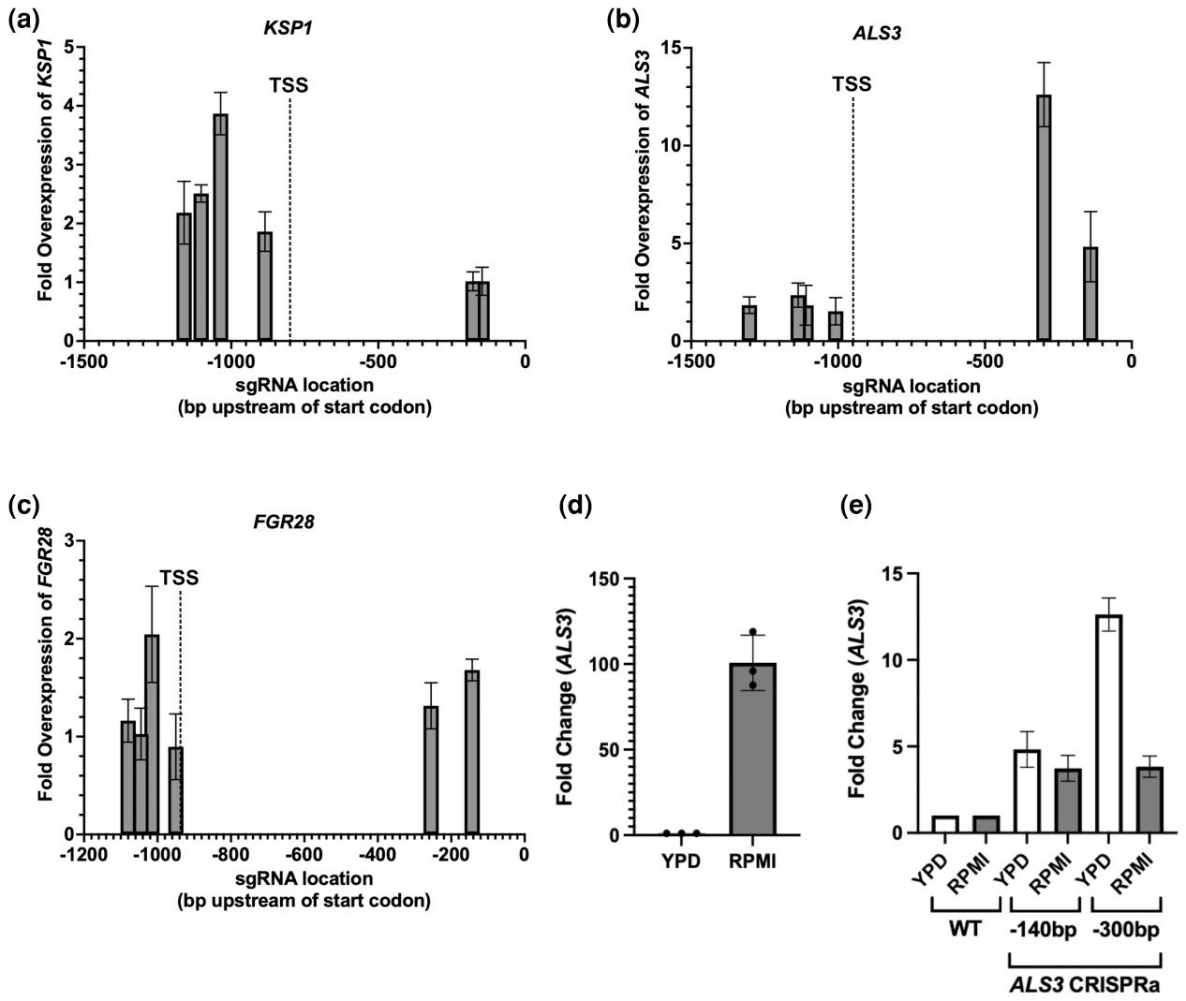
Determining optimal guide-design principles for CRISPRa overexpression in *Candida albicans*. CRISPRa strains were created using 6 different sgRNA molecules designed to target different positions in the promoter region of each of a) *KSP1*, b) *ALS3*, and c) *FGR28* upstream of either the start codon or the predicted dominant TSS. Fold change in the expression of the target gene relative to the housekeeping gene *ACT1* in the experimental strains and the nontargeting CRISPRa control strain was measured with RT-qPCR and compared. Data points represent the mean fold difference and standard deviation in expression of the respective target gene in the CRISPRa strains compared with the nontargeting CRISPRa control (*n* = 3). d) Fold change in the basal expression level of *ALS3* relative to the housekeeping gene *ACT1* in the nontargeting CRISPRa control strain grown in either standard laboratory conditions (YPD at 30°C) or conditions known to favor a high level of *ALS3* expression (RPMI at 37°C) was measured with RT-qPCR. Data represent the mean fold difference and standard deviation in expression of *ALS3* in the nontargeting CRISPRa control strain in both conditions normalized to growth in YPD at 30°C (*n* = 3). e) Fold change in the expression of *ALS3* relative to the housekeeping gene *ACT1* in two CRISPRa strains targeting *ALS3* for overexpression as well as the nontargeting CRISPRa control strain grown in either YPD at 30°C or RPMI at 37°C was measured with RT-qPCR. Data represent the mean fold difference and standard deviation in expression of *ALS3* in all strains in both conditions normalized to the nontargeting CRISPRa control strain (WT; *n* = 3).

We generated CRISPRa plasmids based on these sgRNAs, used these to create *C. albicans* strains, and monitored overexpression of each gene via RT-qPCR. This analysis validated our novel CRISPRa system and its ability to overexpress *C. albicans* genes ∼2- to 12-fold, based on different sgRNA designs and target genes (Fig. 2). We found that in some instances, such as for *KSP1*, the TSS was in fact a better predictor of CRISPRa overexpression, as the most robust overexpression of this gene was observed with sgRNAs targeted upstream of the TSS, while no overexpression was observed based on targeting upstream of the start codon (Fig. 2a). In other instances, such as for *ALS3*, sgRNA targeting upstream of the start codon led to very high levels of overexpression, while sgRNAs targeted to the TSS region led to moderate overexpression (Fig. 2b). For *FGR28*, modest overexpression (∼2-fold) was achieved with sgRNAs targeted to either the start codon or the TSS upstream regions (Fig. 2c), together suggesting that optimal sgRNA design can be predicted based on targeting the TSS and/or start codon, but varies by gene. This data also suggest that our CRISPRa tool may have additional applications in the functional assessment of key transcriptional regulatory regions in *C. albicans*. These differences in sgRNA targeting may be based on differences in overall sgRNA efficiency or may be attributed to inaccurate TSS predictions, or complex promoter dynamics and the preferential use of different TSS regions in different conditions (McMillan *et al.* 2019).

Given that our CRISPRa system has the benefit of exploiting a gene's endogenous promoter for overexpression, we next wanted to determine if our CRISPRa system could be used to promote enhanced expression of genes under conditions that already favored high gene expression. We focused on *ALS3*, where expression levels are known to be significantly enhanced upon growth in conditions that favor filamentous or biofilm growth, such as growth in RPMI media at 37°C (Bahn *et al.* 2007; Ruben *et al.* 2020; Deng *et al.* 2021). We compared basal *ALS3* transcript levels of the *C. albicans* control strain grown in YPD at 30°C compared with RPMI at 37°C via RT-qPCR, and confirmed that *ALS3* exhibits ∼100-fold higher expression in RPMI at 37°C (Fig. 2d). To determine whether our CRISPRa system could promote enhanced overexpression of *ALS3* in these conditions, we monitored *ALS3* expression in two CRISPRa *ALS3* strains (from Fig. 2b) in RPMI at 37°C. We found *ALS3* transcript levels in the CRISPRa strain were further overexpressed ∼4-fold in RPMI at 37°C, despite the existing high level of *ALS3* under these conditions (Fig. 2e). However, while *ALS3* is overexpressed in RPMI via CRISPRa, the degree of overexpression is relatively lower than what occurs in YPD conditions (Fig. 2e). Together this suggests that our CRISPRa system can effectively be used to increase transcription under conditions that already favor high levels of expression of a target gene of interest, but that there may be a maximal level of overexpression possible under these conditions.

### Use of CRISPRa to study antifungal drug susceptibility phenotypes

**Fig. 3.**
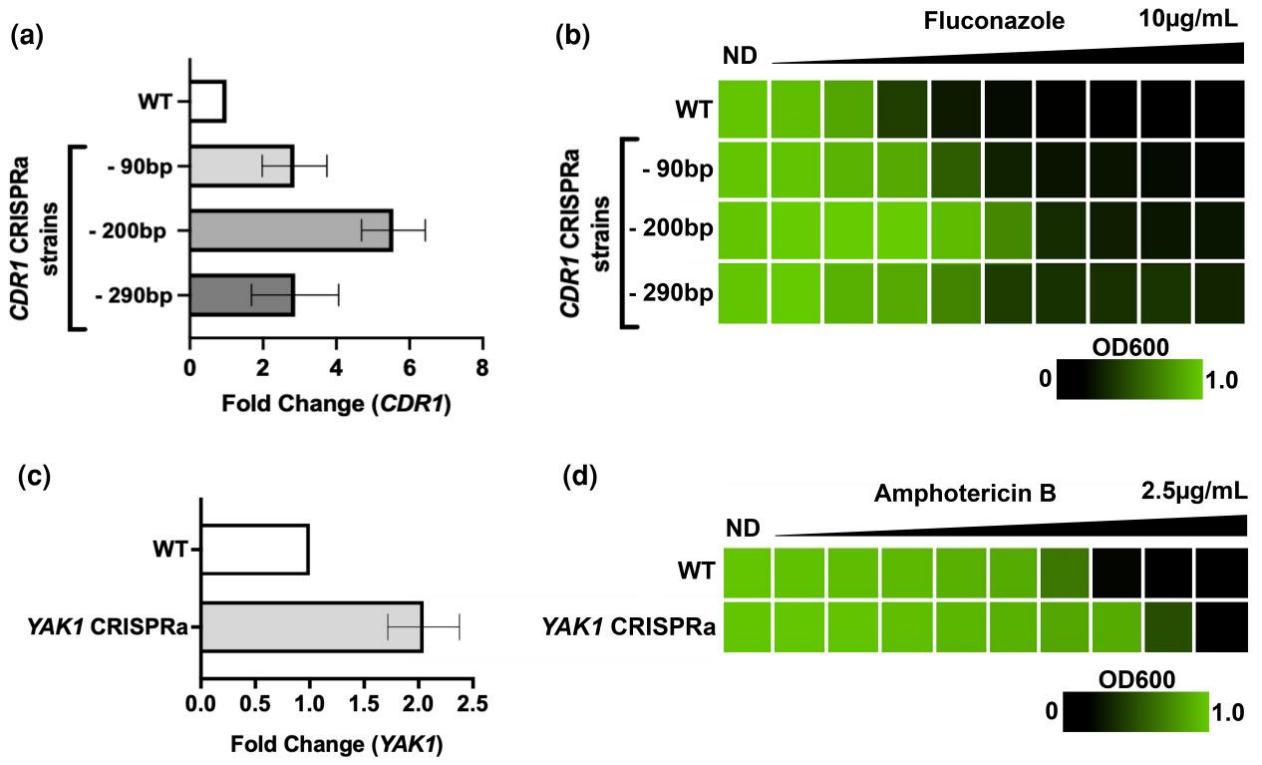
Using CRISPRa to validate antifungal drug *susceptibility* phenotypes. a) Three *C. albicans* CRISPRa strains were created targeting the drug transporter gene *CDR1* for overexpression. Fold change in the expression of *CDR1* relative to the housekeeping gene *ACT1* in the 3 experimental strains and the nontargeting CRISPRa control strain was measured with RT-qPCR. All 3 experimental strains are represented by the position of their sgRNA in the promoter region upstream of the proposed dominant *CDR1* transcriptional start site. Data represent the mean fold difference and standard deviation in expression of *CDR1* in the CRISPRa strains compared with the nontargeting CRISPRa control (WT; *n* = 3). b) The fitness of all 3 CRISPRa strains targeting *CDR1* for overexpression was measured in the presence of the antifungal drug fluconazole with a minimum inhibitory concentration assay. The OD_600_ value of each strain in each concentration of fluconazole was measured to represent growth in differing conditions, where OD_600_ values approaching 1.0 reflect the highest fitness. c) Fold change in the expression of *YAK1* relative to the housekeeping gene *ACT1* in the experimental strain and the nontargeting CRISPRa control strain was measured with RT-qPCR. Data represent the mean fold difference and standard deviation in expression of *YAK1* in the CRISPRa strains compared with the nontargeting CRISPRa control (WT; *n* = 3). d) The fitness of the CRISPRa strain targeting *YAK1* for overexpression was measured in the presence of the antifungal drug amphotericin B with a minimum inhibitory concentration assay. The OD_600_ value of each strain in each concentration of amphotericin B was measured to represent growth in differing conditions, where OD_600_ values approaching 1.0 reflect the highest fitness. ND: no drug.

To validate that the CRISPRa system could recapitulate phenotypes associated with *C. albicans* gene overexpression, we targeted genes with known phenotypes associated with high levels of expression. Initially, we focused on *CDR1*, a well-characterized *C. albicans* gene, which encodes a drug efflux pump (Prasad *et al.* 2015), and whose overexpression is known to play a role in resistance to azole antifungals in laboratory and clinical isolates (Prasad *et al.* 1995; Sanglard *et al.* 1995; White 1997; Marr *et al.* 1998, 2001; Niimi *et al.* 2004; Manoharlal *et al.* 2008). To determine an optimal range in which to target our sgRNA in the *CDR1* promoter, we designed 3 sgRNAs targeting loci ∼90, 200, and 290 bp upstream of the *CDR1* TSS. These sgRNAs were each cloned into our CRISPRa dCas9-VPR backbone and transformed into *C. albicans*. Next, we quantified the expression of *CDR1* in the 3 different fungal strains via RT-qPCR, compared with a control strain containing the CRISPRa plasmid with a nontargeting sgRNA. We found that targeting the dCas9-VPR construct to the *CDR1* promoter enhanced transcription by 2.9- to 5.6-fold (Fig. 3a), indicating that this CRISPRa system is able to drive enhanced levels of gene expression from this target gene of interest. For *CDR1*, we found the highest level of overexpression was obtained by targeting the dCas9-VPR construct ∼200 bp upstream of the TSS (Fig. 3a), which helped further inform the design of sgRNAs for subsequent target genes.

In order to determine if *CDR1*-overexpressing strains had measurable changes in antifungal drug susceptibility phenotypes, we performed MIC assays with these strains in the presence of the antifungal drug fluconazole. We found that strains overexpressing *CDR1* exhibited decreased sensitivity to fluconazole compared with the control strain and that higher levels of overexpression corresponded with lower levels of susceptibility (Fig. 3b). Together, this confirms that our CRISPRa system can be exploited to functionally overexpress target genes of interest, and recapitulate phenotypes associated with high levels of gene expression. Further, targeting dCas9-VPR to different promoter loci leads to variable levels of overexpression, which could be used to study correlations between expression levels and associated phenotypes.

We next sought to determine the ability of our CRISPRa system to be employed to characterize a gene whose overexpression has never been profiled with regard to antifungal drug susceptibility, to our knowledge. We selected the gene *YAK1* as a target for overexpression with CRISPRa. *Candida albicans YAK1* is a predicted serine-threonine protein kinase, inactivation of which renders cells more sensitive to the antifungal amphotericin B (Chen *et al.* 2018). While *YAK1* overexpression has been shown to promote filamentation in *C. albicans* (MacAlpine *et al.* 2021), and there is emerging evidence for a direct relationship between the 2 phenotypes (Sharma *et al.* 2019), the role of overexpressing this factor in antifungal drug susceptibility has not previously been established. We generated a CRISPRa strain overexpressing *YAK1* by ∼2-fold (Fig. 3c) and profiled the ability of this strain to grow in the presence of amphotericin B. We found that the *YAK1* CRISPRa strain was less sensitive to amphotericin B based on MIC testing on this strain compared with the control strain (Fig. 3d). This highlights our capacity to exploit this CRISPRa system to investigate novel phenotypes in *C. albicans*.

### Validation of CRISPRa overexpression targeting fungal pathogenesis traits

**Fig. 4.**
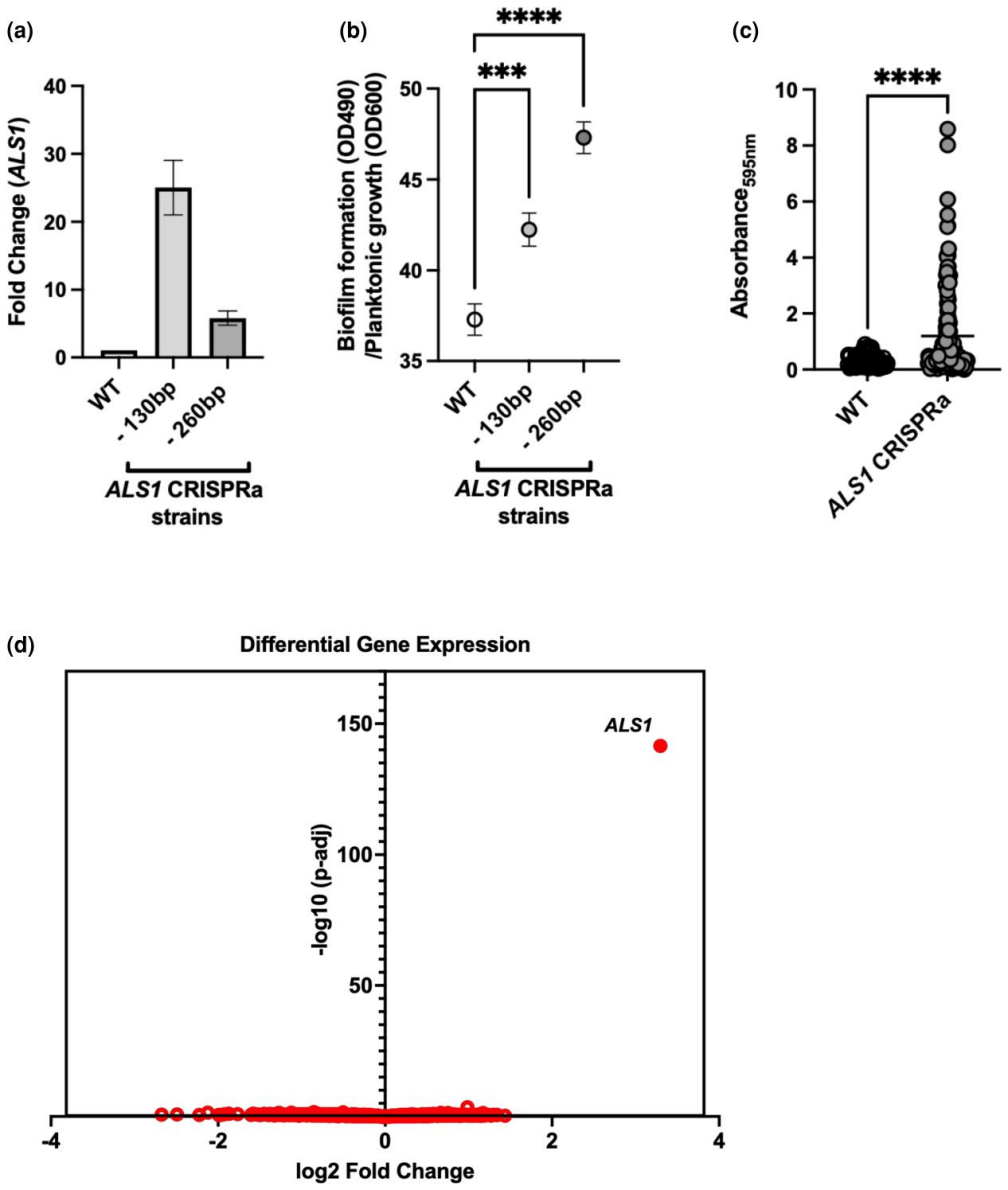
Validation of CRISPRa overexpression and target specificity through targeting fungal pathogenesis traits. a) Two *C. albicans* CRISPRa strains targeting different positions in the promoter region of the adhesin protein gene *ALS1* were created. Fold change in the expression of *ALS1* relative to the housekeeping gene *ACT1* in both experimental strains and the nontargeting CRISPRa control strain was measured with RT-qPCR. Experimental strains are represented by the position of their sgRNA in the promoter region upstream of the proposed dominant *ALS1* transcriptional start site. Data points represent the mean fold difference and standard deviation in expression of *ALS1* in the CRISPRa strains compared with the nontargeting CRISPRa control (WT; *n* = 3). b) Two CRISPRa strains targeting *ALS1* and a nontargeting CRISPRa control strain were grown in the biofilm-inducing media RPMI and left to grow statically at 37°C for 24 h to allow biofilms to form. The robustness of biofilms formed by all strains was elucidated through an XTT reduction assay and measuring of the resulting OD_490_ values before normalizing final values to nonbiofilm-forming planktonic cells. Data points represent the mean and the standard error of the mean (*n* = 72). Differences between groups were tested for significance using Dunnett's multiple comparison test. ****P < 0.0001. ***P < 0.001. c) A *C. albicans* CRISPRa strain overexpressing *ALS1* ∼6-fold and a nontargeting CRISPRa strain were grown in human urine supplemented with fibrinogen and BSA at 37°C for 48 h to simulate a catheterized bladder environment. The robustness of biofilms formed by both strains was measured by reading the OD_595_ value following a crystal violet assay. Data points are combined from 3 independent experiments where *n* = 32 for the nontargeting CRISPRa control and *n* = 48 for the CRISPRa *ALS1* overexpression strain. Differences between groups were tested for significance using the Mann–Whitney *U* test. ****P < 0.0001. d) Global gene expression of a CRISPRa strain overexpressing *ALS1* ∼25-fold, as well as a nontargeting CRISPRa control strain, was measured using RNA-seq. Differential gene expression analysis showed that *ALS1* had a log2-fold change of ∼3.3 in the *ALS1* overexpression strain compared with the CRISPRa control strain, with an adjusted *P*-value of 3.07E-142, and showed no additional transcripts that had differential expression with an adjusted *P*-value of <0.03.

Next, we aimed to validate the CRISPRa system as a means to assess *C. albicans* genes involved in fungal pathogenesis traits. We focused on *ALS1*, a well-characterized *C. albicans* adhesin gene, with important roles in adherence, biofilm formation, and virulence (Fu *et al.* 2002; Kamai *et al.* 2002; Alberti-Segui *et al.* 2004; Zhao *et al.* 2004; Nobile *et al.* 2008; Rosiana *et al.* 2021). Previous work has demonstrated that overexpression of *ALS1* via promoter replacement increases fungal adherence (Fu *et al.* 2002); therefore, we chose to overexpress this gene using our CRISPRa system. We designed and cloned 2 sgRNAs targeting ∼130 and 260 bp upstream of the *ALS1* TSS, and generated *C. albicans ALS1* CRISPRa strains with each of these constructs. We performed RT-qPCR to confirm the overexpression of these strains, which enhanced the expression of *ALS1* by ∼25- and ∼6-fold, respectively (Fig. 4a).To confirm phenotypes associated with overexpression of *ALS1*, we performed two distinct biofilm growth assays that compared the *ALS1* CRISPRa strain to the CRISPRa control strain. In the first assay, we allowed biofilms to establish over 24 h in flat-bottom 96-well polystyrene plates, removed planktonic cells, and quantified the metabolic activity of the biofilm via XTT reduction, relative to the planktonic cell population. We found that the *ALS1* CRISPRa strain formed significantly more robust biofilms, relative to the control strain (*P <* 0.0005; Fig. 4b). Interestingly, biofilm growth was not correlated with *ALS1* overexpression levels, as the strain with ∼25-fold *ALS1* overexpression formed less robust biofilms compared with the strain expressing ∼6-fold more *ALS1*. This suggests that very high levels of *ALS1* may ultimately impair biofilm formation, or that the level of *ALS1* expression varies in the conditions used for RT-qPCR analysis compared with biofilm growth, perhaps due to condition-specific repositioning in the TSS.

Next, to mimic conditions associated with fungal biofilm formation in catheter-associated urinary tract infections (CAUTIs), we used a urine-based assay and analyzed biofilm formation of a *C. albicans* strain overexpressing *ALS1*, relative to its control strain, using crystal violet staining. For this assay, we followed up on the *ALS1* CRISPRa strain with ∼6-fold overexpression of *ALS1*, which had more robust biofilm growth than the control strain (Fig. 4, a and b). The *C. albicans* strains were incubated for 48 h in human urine supplemented with BSA in fibrinogen-coated 96-well plates. Fibrinogen has been shown to be a critical protein for pathogen binding in CAUTIs, by providing a scaffold for biofilm formation and a nutritional source for pathogens (Flores-Mireles *et al.* 2016; La Bella *et al.* 2021; Andersen *et al.* 2022). The *ALS1* CRISPRa overexpression strain showed significantly greater biofilm formation compared with the corresponding wild-type strain (*P* < 0.0001; Fig. 4c). This difference in biofilm formation suggests that *ALS1* promotes binding to fibrinogen and plays a vital role in biofilm formation in urine.

To assess the specificity of this CRISPRa system and ensure that there were no off-target effects from the dCas9–sgRNA complex, we monitored global changes in transcription in the *ALS1* CRISPRa strain via RNA-seq. Since *ALS1* is a member of the large agglutinin-like sequence (ALS) gene family (Hoyer *et al.* 2008; Hoyer and Cota 2016), it may be a likely candidate for any potential off-target effects. We also considered *ALS1* as a strong candidate for this RNA-seq off-target profiling since *ALS1* is not known to be a transcriptional regulator, and therefore its overexpression is unlikely to lead to direct downstream transcriptional alterations that would obscure our analysis of CRISPRa off-target effects. Therefore, we performed RNA-seq on the validated CRISPRa strain overexpressing *ALS1* by ∼25-fold as well as on a control strain containing the dCas9 cassette and a nontargeting sgRNA when grown in standard laboratory conditions. This analysis confirmed the specific overexpression of *ALS1* (log2-fold change ∼3.3 via RNA-seq analysis, adjusted *P*-value 3.07E-142; Fig. 4d, Supplementary Table 2). Five additional transcripts were identified as significantly modulated (adjusted *P*-value < 0.05, absolute log2-fold change > 1; Supplementary Table 2). However, their altered expression was much less significant than *ALS1* (Fig. 4d), and none of them had an adjusted *P*-value of <0.03, differing from the adjusted *P*-value of 3E-142 for *ALS1.* We suspect these additional transcripts’ altered expression is likely unrelated to the CRISPRa system based on the fact that 4/5 genes were down-regulated instead of up-regulated, and none of the genes are in proximity to the *ALS1* locus, nor do they appear to share any sgRNA target similarities in their promoter regions. Together this suggests that this CRISPRa system is likely specific with regards to gene targeting.

## Discussion

Here, we present the development, validation, and application of a single-plasmid-based CRISPRa platform for efficient genetic overexpression in *C. albicans*. We demonstrate the ability of this system to overexpress genes ∼2- to 20-fold, and recapitulate important biological phenotypes associated with genetic overexpression, including biofilm formation and antifungal drug susceptibility. In addition, we assess sgRNA design principles based on predicted TSS, and validate the specificity of this system by monitoring off-target effects via RNA-seq. Together, this work demonstrates an effective, efficient, and specific tool for genetic overexpression in *C. albicans* to enhance the available techniques for genetic modulation in this important fungal pathogen. We believe this tool will have practical application for many researchers, and can be easily applied in other laboratories by following plasmid and strain construction protocols for *C. albicans*–based dCas9 strategies, which we previously described in detail (Wensing and Shapiro 2022). Based on our results, we advise designing and testing 2-3 sgRNAs in regions −100 to −350 bp upstream of both the start codon (ATG) and TSS of the target gene of interest to achieve high levels of overexpression.

Studying genetic overexpression is an important strategy for functional genetic analysis, as overexpressing genes can mimic natural biological states where genes are expressed at high levels. In *C. albicans*, there are numerous scenarios in which the overexpression of genes occurs, including mutations that modify the function of transcriptional regulators and promote the expression of downstream genes (Coste *et al.* 2004, 2006), as well as aneuploidies such as trisomies (Selmecki *et al.* 2006; Forche *et al.* 2009, 2018; Ford *et al.* 2015; Li *et al.* 2015; Anderson *et al.* 2017; Ene *et al.* 2018), isochromosome formation (Selmecki *et al.* 2006, 2008), and copy number variations (Ford *et al.* 2015; Todd and Selmecki 2020). These overexpression events are frequently detected among *C. albicans* clinical isolates (Selmecki *et al.* 2006; Ford *et al.* 2015; Anderson *et al.* 2017) and can play an important role in drug resistance (Selmecki *et al.* 2006, 2008; Li *et al.* 2015; Todd and Selmecki 2020), host adaptation (Forche *et al.* 2018, 2019), and other stress tolerance phenotypes (Yang *et al.* 2019, 2021). For example, amplification of part or all of chromosome 5, via trisomy or isochromosome formation has been tightly linked to the development of resistance to azole antifungals in *C. albicans* via the increased copy number of genes encoding the azole target, Erg11, and the transcriptional regulator of drug efflux, Tac1 (Selmecki *et al.* 2008). The prevalence and importance of these phenomena associated with increased gene expression highlight the relevance of developing new tools to targetedly assess phenotypes associated with genetic overexpression in *C. albicans*.

Harnessing the cell's natural transcriptional machinery to increase gene expression with CRISPRa also leads to a unique set of obstacles. While our RNA-seq results suggest that our CRISPRa system is specific when paired with one sgRNA targeted to *ALS1*, there remains the possibility of off-target effects occurring in different CRISPRa constructs (Zhang *et al.* 2015). Using complementary genetic tools to confirm phenotypes observed from CRISPRa can help mitigate this. One phenomenon shared among many different CRISPRa activation domains, including VPR, is a reduced capacity to induce overexpression in genes that are already expressed at a high levels (Chavez *et al.* 2016). This may explain why we achieved only modest overexpression of *YAK1*, the gene with the highest basal expression level of all genes tested, but also why a ∼2-fold change in transcript abundance still resulted in a clear decrease in susceptibility to amphotericin B (Fig. 3d). Incidentally, the gene that we achieved the lowest overexpression with across all of our testing, *FGR28*, had the lowest basal expression (Supplementary Table 3). However, efficiency of the sgRNA molecule and its exact position in the target region of the gene of interest, as well as a host of promoter dynamics, can also influence CRISPR-Cas9 efficacy (Radzisheuskaya *et al.* 2016; Cámara *et al.* 2020; Lu and Lin 2021). This illustrates why multiple sgRNAs need to be designed when targeting a gene with CRISPRa to maximize successful overexpression of the target gene. While the rapid and inexpensive nature of CRISPRa strain construction justifies the need to design several sgRNA molecules for a given gene, limitations resulting from the specific “NGG” PAM recognition site by *Streptococcus pyogenes* Cas9 proteins leads to the restraint that, on rare occasions, it may not be possible to produce any efficient sgRNA molecules in a suitable region of the promoter (Uthayakumar *et al.* 2021). It may therefore be prudent to further expand the CRISPR toolbox in *C. albicans* by introducing novel Cas9 variants with differing PAM recognition sites, or by constructing novel CRISPR systems exploiting alternative Cas proteins with different and broader PAM recognition capacities, offering the ability to target regions in the genome that cannot be manipulated with current CRISPR-Cas9 tools (Hu *et al.* 2018; Ciurkot *et al.* 2021).

Alternative overexpression strategies in *C. albicans* that involve introducing an extra copy of a gene of interest or introducing an alternative promoter have been used to construct and screen libraries composed of hundreds and even thousands of protein-coding genes (Rai *et al.* 2021). These studies have revealed novel insights into gene function related to morphogenesis (Chauvel *et al.* 2012), cell cycle progression (Jaitly *et al.* 2022), drug tolerance (Delarze *et al.* 2020), and biofilm formation (Cabral *et al.* 2014). Previous comparisons between CRISPRa and ORF-based overexpression libraries in other species have highlighted the potential for these complementary strategies to both confirm findings and unveil distinct genetic hits (Sanson *et al.* 2018). CRISPRa offers the advantage of being high throughput and easily scalable into a large-scale pooled format, and mutant libraries in different strain backgrounds can therefore be quickly generated (Sanjana 2017; Momen-Roknabadi *et al.* 2020). In contrast, ORF-based overexpression strategies may be preferable if attempting to overexpress only a single transcript variant or isoform (La Russa and Qi 2015), and promoter replacement strategies may be more valuable to overexpress diverse genes to similar levels. Both of these strategies would be advantageous as well in rare instances when a target sequence lacks a PAM site. Non-CRISPR overexpression libraries previously assembled in *C. albicans* have the potential weakness of missing phenotypes if the level of expression achieved in mutant strains is too low (Znaidi *et al.* 2018). The ability of our system to cause upwards of 25-fold overexpression of gene targets is thus an important feature, and future iterations of this CRISPRa system could also exploit an inducible dCas9-VPR construct for tunability of gene expression. Furthermore, phenotypes in mutant strains may only be observable if the level of overexpression closely mimics the level of upregulation that naturally occurs in the cell in a given condition (Znaidi *et al.* 2018). That our CRISPRa system exploits the endogenous promoter to drive upregulation may thus be critical in understanding natural overexpression mechanisms and regulatory circuitry that other systems are not capable of studying.

While large-scale CRISPRa libraries have not been generated in *C. albicans*, their application in other organisms, namely mammalian cell lines, has enabled important discoveries in cancer biology, stem cell reprogramming, cellular fitness, and drug resistance (Gilbert *et al.* 2014; Konermann *et al.* 2015; Horlbeck *et al.* 2016; Joung *et al.* 2017; Jost *et al.* 2017; Kampmann 2018; Sanson *et al.* 2018; Yang *et al.* 2019). The development of CRISPRa systems in *C. albicans* could enable the development of larger overexpression libraries for functional genomic screens to complement existing fungal gene deletion and depletion libraries (Roemer *et al.* 2003; Homann *et al.* 2009; Blankenship *et al.* 2010; Noble *et al.* 2010), that have been subject to robust screening and analysis under diverse conditions (Gervais *et al.* 2021) to reveal critical new insight into fungal biology (Lohse *et al.* 2016; Hossain *et al.* 2020), pathogenesis (Noble *et al.* 2010; Nobile *et al.* 2012; Ryan *et al.* 2012; O’Meara *et al.* 2015; Lee *et al.* 2016; Hossain *et al.* 2020), and mechanisms of drug and stress tolerance (Motaung *et al.* 2017; Mount *et al.* 2018; Caplan *et al.* 2018; Fu *et al.* 2021). The application of similar CRISPRa systems to other fungal organisms would lend further insight into fungal biology across this kingdom. Many CRISPR tools have been developed in a diversity of fungal organisms (Muñoz *et al.* 2019; Cai *et al.* 2019; Román, Prieto, *et al.* 2019; Schuster and Kahmann 2019; Morio *et al.* 2020; Ouedraogo and Tsang 2020; Shan *et al.* 2021), including numerous non-*albicans Candida* species (Enkler *et al.* 2016; Lombardi *et al.* 2017, 2019; Zoppo *et al.* 2019; Maroc and Fairhead 2019; Uthayakumar *et al.* 2021; Ennis *et al.* 2021; Santana and O’Meara 2021), and other prominent pathogens such as *Cryptococcus neoformans* (Fan and Lin 2018; Wang 2018; Huang *et al.* 2021) and *Aspergillus fumigatus* (Fuller *et al.* 2015; Zhang *et al.* 2016; Al Abdallah *et al.* 2018; van Rhijn *et al.* 2020). These CRISPR platforms could be adapted to similar CRISPRa tools in these organisms, with many possible applications for the analysis of pathogen biology or drug target identification (Li *et al.* 2004; Luesch *et al.* 2005; Smith *et al.* 2010; Arnoldo *et al.* 2014; Begolo *et al.* 2014).

Genetic overexpression systems also have many useful applications for industrially important fungi, as targeted genetic overexpression can be exploited for metabolic engineering to enhance the production of commercially important metabolites (Donohoue *et al.* 2018; Zhao *et al.* 2018; Ouedraogo and Tsang 2020; Jiang *et al.* 2021). CRISPR techniques have already been adapted to numerous industrially relevant fungi (Nødvig *et al.* 2015; Kwon *et al.* 2019; Jiang *et al.* 2021; Wilson and Harrison 2021), and CRISPRa has recently been optimized for use in the filamentous fungi *Aspergillus nidulans* and *Penicillium rubens* (Roux *et al.* 2020; Mózsik *et al.* 2021). These CRISPRa systems were applied to activate transcriptionally silent biosynthetic gene clusters in these filamentous fungi, with important applications for the discovery of novel bioactive molecules (Roux *et al.* 2020; Mózsik *et al.* 2021). As many *Candida* species play important industrial roles in manufacturing metabolites for food and pharmaceutical industries, as well as remediating wastewater via hydrocarbon degradation (Joo *et al.* 2008; Gargouri *et al.* 2015; Kieliszek *et al.* 2017; Payen and Thompson 2019; García-Béjar *et al.* 2020), species-specific adaptations to the CRISPRa tool presented here could produce optimized *Candida* strains for valuable biomanufacturing and bioremediation applications.

## Supplementary Material

jkac301_Supplementary_Data

## Data Availability

The *C. albicans* CRISPRa plasmid backbone was deposited to Addgene as plasmid number #182707. RNA-seq data were deposited in NCBI GEO repository (accession number GSE201904; https://www.ncbi.nlm.nih.gov/geo/query/acc.cgi?acc=GSE201904). Supplemental material available at G3 online.

## Notes

*Communicating editor: J. Heitman*

## References

[jkac301-B1] Adli M . The CRISPR tool kit for genome editing and beyond. Nat Commun. 2018;9(1):1911. doi:10.1038/s41467-018-04252-2 29765029 PMC5953931

[jkac301-B2] Al Abdallah Q , Souza ACO, Martin-Vicente A, Ge W, Fortwendel JR. Whole-genome sequencing reveals highly specific gene targeting by *in vitro* assembled Cas9-ribonucleoprotein complexes in *Aspergillus fumigatus*. Fungal Biol Biotechnol. 2018;5(1):11. doi:10.1186/s40694-018-0057-2 29992034 PMC5987418

[jkac301-B3] Alberti-Segui C , Morales AJ, Xing H, Kessler MM, Willins DA, et al Identification of potential cell-surface proteins in *Candida albicans* and investigation of the role of a putative cell-surface glycosidase in adhesion and virulence. Yeast. 2004;21(4):285–302. doi:10.1002/yea.1061 15042589

[jkac301-B4] Andersen MJ , Fong C, La Bella AA, Molina JJ, Molesan A, et al Inhibiting host-protein deposition on urinary catheters reduces associated urinary tract infections. eLife. 2022;11:e75798. doi:10.7554/eLife.75798 35348114 PMC8986317

[jkac301-B5] Anderson MZ , Saha A, Haseeb A, Bennett RJ. A chromosome 4 trisomy contributes to increased fluconazole resistance in a clinical isolate of *Candida albicans*. Microbiology. 2017;163(6):856–865. doi:10.1099/mic.0.000478 28640746 PMC5737213

[jkac301-B6] Arendrup MC , Patterson TF. Multidrug-resistant *Candida*: epidemiology, molecular mechanisms, and treatment. J Infect Dis. 2017;216(suppl 3):S445–S451. doi:10.1093/infdis/jix131 28911043

[jkac301-B7] Arnoldo A , Kittanakom S, Heisler LE, Mak AB, Shukalyuk AI, et al A genome scale overexpression screen to reveal drug activity in human cells. Genome Med. 2014;6(4):32. doi:10.1186/gm549 24944581 PMC4062067

[jkac301-B8] Bahn Y-S , Molenda M, Staab JF, Lyman CA, Gordon LJ, et al Genome-wide transcriptional profiling of the cyclic AMP-dependent signaling pathway during morphogenic transitions of *Candida albicans*. Eukaryot Cell. 2007;6(12):2376–2390. doi:10.1128/EC.00318-07 17951520 PMC2168245

[jkac301-B9] Begolo D , Erben E, Clayton C. Drug target identification using a trypanosome overexpression library. Antimicrob Agents Chemother. 2014;58 (10):6260–6264. doi:10.1128/AAC.03338-14 25049244 PMC4187942

[jkac301-B10] Benedict K , Jackson BR, Chiller T, Beer KD. Estimation of direct healthcare costs of fungal diseases in the United States. Clin Infect Dis. 2018;68(11):1791–1797. doi:10.1093/cid/ciy776 PMC6409199 30204844

[jkac301-B156] Blankenship JR , Fanning S, Hamaker JJ, Mitchell AP. An extensive circuitry for cell wall regulation in *Candida albicans*. PLoS Pathogens. 2010;6:e1000752. doi:10.1371/journal.ppat.1000752 20140194 PMC2816693

[jkac301-B11] Bock C , Datlinger P, Chardon F, Coelho MA, Dong MB, et al High-content CRISPR screening. Nat Rev Methods Primers. 2022;2(1):1–1. doi:10.1038/s43586-021-00093-4 PMC10200264 37214176

[jkac301-B12] Bongomin F , Gago S, Oladele R, Denning D. Global and multi-national prevalence of fungal diseases—estimate precision. J Fungi (Basel). 2017;3(4):57. doi:10.3390/jof3040057 29371573 PMC5753159

[jkac301-B13] Cabral V , Znaidi S, Walker LA, Martin-Yken H, Dague E, et al Targeted changes of the cell wall proteome influence *Candida albicans* ability to form single- and multi-strain biofilms. PLoS Pathog. 2014;10(12):e1004542. doi:10.1371/journal.ppat.1004542 PMC4263760 25502890

[jkac301-B14] Cai P , Gao J, Zhou Y. CRISPR-mediated genome editing in non-conventional yeasts for biotechnological applications. Microb Cell Fact. 2019;18(1):63. doi:10.1186/s12934-019-1112-2 30940138 PMC6444819

[jkac301-B15] Cámara E , Lenitz I, Nygård Y. A CRISPR activation and interference toolkit for industrial *Saccharomyces cerevisiae* strain KE6–12. Sci Rep. 2020;10(1):14605. doi:10.1038/s41598-020-71648-w 32884066 PMC7471924

[jkac301-B158] Caplan T , Polvi EJ, Xie JL, Buckhalter S, Leach MD, et al Functional genomic screening reveals core modulators of echinocandin stress responses in *Candida albicans*. Cell Rep. 2018;23:2292–2298. doi:10.1016/j.celrep.2018.04.084 29791841

[jkac301-B16] Chauvel M , Nesseir A, Cabral V, Znaidi S, Goyard S, et al A versatile overexpression strategy in the pathogenic yeast *Candida albicans*: identification of regulators of morphogenesis and fitness. PLoS One. 2012;7(9):e45912. doi:10.1371/journal.pone.0045912 23049891 PMC3457969

[jkac301-B17] Chavez A , Scheiman J, Vora S, Pruitt BW, Tuttle M, et al Highly efficient Cas9-mediated transcriptional programming. Nat Methods. 2015;12(4):326–328. doi:10.1038/nmeth.3312 25730490 PMC4393883

[jkac301-B18] Chavez A , Tuttle M, Pruitt BW, Ewen-Campen B, Chari R, et al Comparison of Cas9 activators in multiple species. Nat Methods. 2016;13(7):563–567. doi:10.1038/nmeth.3871 27214048 PMC4927356

[jkac301-B19] Chen Y , Mallick J, Maqnas A, Sun Y, Choudhury BI, et al Chemogenomic profiling of the fungal pathogen *Candida albicans*. Antimicrob Agents Chemother. 2018;62(2):e02365-17. doi:10.1128/AAC.02365-17 PMC5786791 29203491

[jkac301-B20] Ciurkot K , Gorochowski TE, Roubos JA, Verwaal R. Efficient multiplexed gene regulation in *Saccharomyces cerevisiae* using dCas12a. Nucleic Acids Res. 2021;49(13):7775–7790. doi:10.1093/nar/gkab529 34197613 PMC8287914

[jkac301-B21] Coste AT , Karababa M, Ischer F, Bille J, Sanglard D. *TAC1*, Transcriptional activator of *CDR* genes, is a new transcription factor involved in the regulation of *Candida albicans* ABC transporters *CDR1* and *CDR2*. Eukaryot Cell. 2004;3(6):1639–1652. doi:10.1128/EC.3.6.1639-1652.2004 15590837 PMC539021

[jkac301-B22] Coste A , Turner V, Ischer F, Morschhäuser J, Forche A, et al A mutation in Tac1p, a transcription factor regulating *CDR1* and *CDR2*, is coupled with loss of heterozygosity at chromosome 5 to mediate antifungal resistance in *Candida albicans*. Genetics. 2006;172(4):2139–2156. doi:10.1534/genetics.105.054767 16452151 PMC1456413

[jkac301-B23] Delarze E , Brandt L, Trachsel E, Patxot M, Pralong C, et al Identification and characterization of mediators of fluconazole tolerance in *Candida albicans*. Front Microbiol. 2020;11:591140. doi:10.3389/fmicb.2020.591140 33262748 PMC7686038

[jkac301-B24] Delgado ML , Luisa Delgado M, Luisa Gil M, Gozalbo D. *Candida albicans TDH3* gene promotes secretion of internal invertase when expressed in *Saccharomyces cerevisiae* as a glyceraldehyde-3-phosphate dehydrogenase-invertase fusion protein. Yeast. 2003;20(8):713–722. doi:10.1002/yea.993 12794932

[jkac301-B25] Deng K , Jiang W, Jiang Y, Deng Q, Cao J, et al ALS3 Expression as an indicator for *Candida albicans* biofilm formation and drug resistance. Front Microbiol. 2021;12:655242. doi:10.3389/fmicb.2021.655242 33995316 PMC8117015

[jkac301-B26] Dominguez AA , Lim WA, Qi LS. Beyond editing: repurposing CRISPR-Cas9 for precision genome regulation and interrogation. Nat Rev Mol Cell Biol. 2016;17(1):5–15. doi:10.1038/nrm.2015.2 26670017 PMC4922510

[jkac301-B27] Donohoue PD , Barrangou R, May AP. Advances in industrial biotechnology using CRISPR-Cas systems. Trends Biotechnol. 2018;36(2):134–146. doi:10.1016/j.tibtech.2017.07.007 28778606

[jkac301-B28] Eckert SE , Mühlschlegel FA. Promoter regulation in *Candida albicans* and related species. FEMS Yeast Res. 2009;9(1):2–15. doi:10.1111/j.1567-1364.2008.00455.x 19054124

[jkac301-B157] Edgar R , Domrachev M, Lash AE. Gene expression omnibus: NCBI gene expression and hybridization array data repository. Nucleic Acids Res. 2002;30:207–210. doi:10.1093/nar/30.1.207 11752295 PMC99122

[jkac301-B29] Ene IV , Farrer RA, Hirakawa MP, Agwamba K, Cuomo CA, et al Global analysis of mutations driving microevolution of a heterozygous diploid fungal pathogen. Proc Natl Acad Sci U S A. 2018;115(35):8688–8697. doi:10.1073/pnas.1805234115 30150418 PMC6140516

[jkac301-B30] Engler C , Kandzia R, Marillonnet S. A one pot, one step, precision cloning method with high throughput capability. PLoS One. 2008;3(11):e3647. doi:10.1371/journal.pone.0003647 18985154 PMC2574415

[jkac301-B31] Enkler L , Richer D, Marchand AL, Ferrandon D, Jossinet F. Genome engineering in the yeast pathogen *Candida glabrata* using the CRISPR-Cas9 system. Sci Rep. 2016;6(1):35766. doi:10.1038/srep35766 27767081 PMC5073330

[jkac301-B32] Ennis CL , Hernday AD, Nobile CJ. A markerless CRISPR-mediated system for genome editing in *Candida auris* reveals a conserved role for Cas5 in the caspofungin response. Microbiol Spectr. 2021;9(3):e0182021. doi:10.1128/Spectrum.01820-21 PMC8567271 34730409

[jkac301-B33] Fan Y , Lin X. Multiple applications of a transient CRISPR-Cas9 coupled with electroporation (TRACE) system in the *Cryptococcus neoformans* species complex. Genetics. 2018;208(4):1357–1372. doi:10.1534/genetics.117.300656 29444806 PMC5887135

[jkac301-B34] Fisher MC , Gurr SJ, Cuomo CA, Blehert DS, Jin H, et al Threats posed by the fungal kingdom to humans, wildlife, and agriculture. mBio. 2020;11:e00449-20. doi:10.1128/mBio.00449-20 10.1128/mBio.00449-20 PMC7403777 32371596

[jkac301-B35] Flores-Mireles AL , Walker JN, Bauman TM, Potretzke AM, Schreiber HL 4th, et al Fibrinogen release and deposition on urinary catheters placed during urological procedures. J Urol. 2016;196(2):416–421. doi:10.1016/j.juro.2016.01.100 26827873 PMC4965327

[jkac301-B36] Flowers SA , Barker KS, Berkow EL, Toner G, Chadwick SG, et al Gain-of-function mutations in *UPC2* are a frequent cause of *ERG11* upregulation in azole-resistant clinical isolates of *Candida albicans*. Eukaryot Cell. 2012;11(10):1289–1299. doi:10.1128/EC.00215-12 22923048 PMC3485914

[jkac301-B37] Forche A , Cromie G, Gerstein AC, Solis NV, Pisithkul T, et al Rapid phenotypic and genotypic diversification after exposure to the oral host niche in *Candida albicans*. Genetics. 2018;209(3):725–741. doi:10.1534/genetics.118.301019 29724862 PMC6028260

[jkac301-B38] Forche A , Magee PT, Selmecki A, Berman J, May G. Evolution in *Candida albicans* populations during a single passage through a mouse host. Genetics. 2009;182(3):799–811. doi:10.1534/genetics.109.103325 19414562 PMC2710160

[jkac301-B39] Forche A , Solis NV, Swidergall M, Thomas R, Guyer A, et al Selection of *Candida albicans* trisomy during oropharyngeal infection results in a commensal-like phenotype. PLoS Genet. 2019;15(5):e1008137. doi:10.1371/journal.pgen.1008137 PMC6538192 31091232

[jkac301-B40] Ford CB , Funt JM, Abbey D, Issi L, Guiducci C, et al The evolution of drug resistance in clinical isolates of *Candida albicans*. eLife. 2015;4:e00662. doi:10.7554/eLife.00662 25646566 10.7554/eLife.00662 PMC4383195

[jkac301-B41] Fu Y , Ibrahim AS, Sheppard DC, Chen Y-C, French SW, et al *Candida albicans* Als1p: an adhesin that is a downstream effector of the *EFG1* filamentation pathway. Mol Microbiol. 2002;44(1):61–72. doi:10.1046/j.1365-2958.2002.02873.x 11967069

[jkac301-B42] Fu Y , Luo G, Spellberg BJ, Edwards JE Jr, Ibrahim AS. Gene overexpression/suppression analysis of candidate virulence factors of *Candida albicans*. Eukaryot Cell. 2008;7(3):483–492. doi:10.1128/EC.00445-07 18178776 PMC2268510

[jkac301-B43] Fuller KK , Chen S, Loros JJ, Dunlap JC. Development of the CRISPR/Cas9 system for targeted gene disruption in *Aspergillus fumigatus*. Eukaryot Cell. 2015;14(11):1073–1080. doi:10.1128/EC.00107-15 26318395 PMC4621320

[jkac301-B161] Fu C , Zhang X, Veri AO, Iyer KR, Lash E, et al Leveraging machine learning essentiality predictions and chemogenomic interactions to identify antifungal targets. Nat Commun. 2021;12:6497. doi:10.1038/s41467-021-26850-3 PMC8586148 34764269

[jkac301-B44] García-Béjar B , Arévalo-Villena M, Guisantes-Batan E, Rodríguez-Flores J, Briones A. Study of the bioremediatory capacity of wild yeasts. Sci Rep. 2020;10(1):11265. doi:10.1038/s41598-020-68154-4 32647290 PMC7347596

[jkac301-B45] Gargouri B , Mhiri N, Karray F, Aloui F, Sayadi S. Isolation and characterization of hydrocarbon-degrading yeast strains from petroleum contaminated industrial wastewater. Biomed Res Int. 2015;2015:929424. doi:10.1155/2015/929424 26339653 PMC4538589

[jkac301-B46] Geddes-McAlister J , Shapiro RS. New pathogens, new tricks: emerging, drug-resistant fungal pathogens and future prospects for antifungal therapeutics. Ann N Y Acad Sci. 2018;1435(1):57–78. doi:10.1111/nyas.13739 29762860

[jkac301-B47] Gerami-Nejad M , Zacchi LF, McClellan M, Matter K, Berman J. Shuttle vectors for facile gap repair cloning and integration into a neutral locus in *Candida albicans*. Microbiology. 2013;159 (Pt 3):565–579. doi:10.1099/mic.0.064097-0 23306673 PMC3709822

[jkac301-B163] Gervais NC , Halder V, Shapiro RS. A data library of *Candida albicans* functional genomic screens. FEMS Yeast Res. 2021;21:foab060. doi:10.1093/femsyr/foab060 10.1093/femsyr/foab060 34864983

[jkac301-B164] Gilbert LA , Horlbeck MA, Adamson B, Villalta JE, Chen Y, et al Genome-scale CRISPR-mediated control of gene repression and activation. Cell. 2014;159:647–661. doi:10.1016/j.cell.2014.09.029 25307932 PMC4253859

[jkac301-B48] Gow NAR , van de Veerdonk FL, Brown AJP, Netea MG. *Candida albicans* morphogenesis and host defence: discriminating invasion from colonization. Nat Rev Microbiol. 2011;10:112–122. doi:10.1038/nrmicro2711 22158429 10.1038/nrmicro2711 PMC3624162

[jkac301-B49] Halder V , Porter CBM, Chavez A, Shapiro RS. Design, execution, and analysis of CRISPR–Cas9-based deletions and genetic interaction networks in the fungal pathogen *Candida albicans*. Nat Protoc. 2019;14(3):955–975. doi:10.1038/s41596-018-0122-6 30737491

[jkac301-B50] Hardwick JM , Tse L, Applegren N, Nicholas J, Veliuona MA. The Epstein-Barr virus R transactivator (Rta) contains a complex, potent activation domain with properties different from those of VP16. J Virol. 1992;66(9):5500–5508. doi:10.1128/jvi.66.9.5500-5508.1992 1323708 PMC289108

[jkac301-B465] Homann OR , Dea J, Noble SM, Johnson D. A phenotypic profile of the *Candida albicans* regulatory network. PLoS Genet. 2009;5:e1000783. doi:10.1371/journal.pgen.1000783 PMC2790342 20041210

[jkac301-B166] Horlbeck MA , Gilbert LA, Villalta JE, Adamson B, Pak RA, et al Compact and highly active next-generation libraries for CRISPR-mediated gene repression and activation. eLife. 2016;5:e19760. doi:10.7554/eLife.19760 27661255 PMC5094855

[jkac301-B167] Hossain S , Veri AO, Cowen LE. The proteasome governs fungal morphogenesis via functional connections with Hsp90 and cAMP-protein kinase a signaling. mBio. 2020;11:e00290-20. doi:10.1128/mBio.00290-20 32317319 PMC7175089

[jkac301-B51] Hoyer LL , Cota E. *Candida albicans* agglutinin-like sequence (als) family vignettes: a review of als protein structure and function. Front Microbiol. 2016;7:280. doi:10.3389/fmicb.2016.00280 27014205 PMC4791367

[jkac301-B52] Hoyer LL , Green CB, Oh S-H, Zhao X. Discovering the secrets of the *Candida albicans* agglutinin-like sequence (ALS) gene family–a sticky pursuit. Med Mycol. 2008;46(1):1–15. doi:10.1080/13693780701435317 17852717 PMC2742883

[jkac301-B53] Hu JH , Miller SM, Geurts MH, Tang W, Chen L, et al Evolved Cas9 variants with broad PAM compatibility and high DNA specificity. Nature. 2018;556(7699):57–63. doi:10.1038/nature26155 29512652 PMC5951633

[jkac301-B54] Huang MY , Joshi MB, Boucher MJ, Lee S, Loza LC, et al Short homology-directed repair using optimized Cas9 in the pathogen *Cryptococcus neoformans* enables rapid gene deletion and tagging. Genetics. 2021;220(1):iyab180. doi:10.1093/genetics/iyab180 PMC8733451 34791226

[jkac301-B55] Huang MY , Mitchell AP. Marker recycling in *Candida albicans* through CRISPR-Cas9-induced marker excision. mSphere. 2017;2:e00050-17. doi:10.1128/mSphere.00050-17 10.1128/mSphere.00050-17 PMC5352831 28317025

[jkac301-B56] Jaitly P , Legrand M, Das A, Patel T, Chauvel M, et al A phylogenetically-restricted essential cell cycle progression factor in the human pathogen *Candida albicans*. Nat Commun. 2022;13(1):4256. doi:10.1038/s41467-022-31980-3 35869076 PMC9307598

[jkac301-B57] Jensen MK . Design principles for nuclease-deficient CRISPR-based transcriptional regulators. FEMS Yeast Res. 2018;18(4):foy039. doi:10.1093/femsyr/foy039 PMC5932555 29726937

[jkac301-B58] Jiang C , Lv G, Tu Y, Cheng X, Duan Y, et al Applications of CRISPR/Cas9 in the synthesis of secondary metabolites in filamentous fungi. Front Microbiol. 2021;12:638096. doi:10.3389/fmicb.2021.638096 33643273 PMC7905030

[jkac301-B59] Joo H-S , Ndegwa PM, Shoda M, Phae C-G. Bioremediation of oil-contaminated soil using *Candida catenulata* and food waste. Environ Pollut. 2008;156(3):891–896. doi:10.1016/j.envpol.2008.05.026 18620787

[jkac301-B159] Jost, M, Chen Y, Gilbert LA, Horlbeck MA, Krenning L, et al Combined CRISPRi/a-based chemical genetic screens reveal that rigosertib is a microtubule-destabilizing agent. Mol Cell. 2017;68:210–223.e6. doi:10.1016/j.molcel.2017.09.012 28985505 10.1016/j.molcel.2017.09.012 PMC5640507

[jkac301-B160] Joung J , Engreitz JM, Konermann S, Abudayyeh OO, Verdine Vk, et al Genome-scale activation screen identifies a lncRNA locus regulating a gene neighbourhood. Nature. 2017;548:343–346. doi:10.1038/nature23451 28792927 PMC5706657

[jkac301-B60] Kamai Y , Kubota M, Kamai Y, Hosokawa T, Fukuoka T, et al Contribution of *Candida albicans ALS1* to the pathogenesis of experimental oropharyngeal candidiasis. Infect Immun. 2002;70(9):5256–5258. doi:10.1128/IAI.70.9.5256-5258.2002 12183577 PMC128218

[jkac301-B61] Kampmann M . CRISPRi and CRISPRa screens in mammalian cells for precision biology and medicine. ACS Chem Biol. 2018;13(2):406–416. doi:10.1021/acschembio.7b00657 29035510 PMC5886776

[jkac301-B62] Kieliszek M , Kot AM, Bzducha-Wróbel A, BŁażejak S, Gientka I, et al Biotechnological use of *Candida* yeasts in the food industry: a review. Fungal Biol Rev. 2017;31(4):185–198. doi:10.1016/j.fbr.2017.06.001

[jkac301-B162] Konermann S , Brigham MD, Trevino AE, Joung J, Abudayyeh OO, et al Genome-scale transcriptional activation by an engineered CRISPR-Cas9 complex. Nature. 2015;517:583–588. doi:10.1038/nature14136 25494202 PMC4420636

[jkac301-B63] Kwon MJ , Schütze T, Spohner S, Haefner S, Meyer V. Practical guidance for the implementation of the CRISPR genome editing tool in filamentous fungi. Fungal Biol Biotechnol. 2019;6(1):15. doi:10.1186/s40694-019-0079-4 31641526 PMC6796461

[jkac301-B64] La Bella AA , Andersen MJ, Gervais NC, Molina JJ, Molesan A, et al Catheterized bladder environment induces *Candida albicans* hyphal formation and promotes colonization and persistence through Als1. bioRxiv. 2021. doi:10.1101/2021.06.01.446547, 9 May 2022, preprint: not peer reviewed.

[jkac301-B65] La Russa MF , Qi LS. The new state of the art: Cas9 for gene activation and repression. Mol Cell Biol. 2015;35:3800–3809. doi:10.1128/MCB.00512-15 26370509 10.1128/MCB.00512-15 PMC4609748

[jkac301-B165] Lee JA , Robbins N, Xie JL, Ketela T, Cowen LE. Functional genomic analysis of *Candida albicans* adherence reveals a key role for the Arp2/3 complex in cell wall remodelling and biofilm formation. PLoS Genet. 2016;12:e1006452. doi:10.1371/journal.pgen.1006452 27870871 PMC5147769

[jkac301-B66] Legrand M , Bachellier-Bassi S, Lee KK, Chaudhari Y, Tournu H, et al Generating genomic platforms to study *Candida albicans* pathogenesis. Nucleic Acids Res. 2018;46(14):6935–6949. doi:10.1093/nar/gky594 29982705 PMC6101633

[jkac301-B67] Li X , Yang F, Li D, Zhou M, Wang X, et al Trisomy of chromosome R confers resistance to triazoles in *Candida albicans*. Med Mycol. 2015;53(3):302–309. doi:10.1093/mmy/myv002 25792759

[jkac301-B68] Li X , Zolli-Juran M, Cechetto JD, Daigle DM, Wright GD, et al Multicopy suppressors for novel antibacterial compounds reveal targets and drug efflux susceptibility. Chem Biol. 2004;11(10):1423–1430. doi:10.1016/j.chembiol.2004.08.014 15489169

[jkac301-B69] Lohberger A , Coste AT, Sanglard D. Distinct roles of *Candida albicans* drug resistance transcription factors *TAC1*, *MRR1*, and *UPC2* in virulence. Eukaryot Cell. 2014;13(1):127–142. doi:10.1128/EC.00245-13 24243794 PMC3910953

[jkac301-B168] Lohse MB , Ene IV, Craik VB, Hernday AD, Mancera E, et al Systematic genetic screen for transcriptional regulators of the *Candida albicans* white-opaque switch. Genetics. 2016;203:1679–1692. doi:10.1534/genetics.116.190645 27280690 PMC4981270

[jkac301-B70] Lombardi L , Oliveira-Pacheco J, Butler G. Plasmid-based CRISPR-Cas9 gene editing in multiple *Candida* species. mSphere. 2019;4(2):e00125–19. doi:10.1128/mSphere.00125-19 PMC6416365 30867327

[jkac301-B71] Lombardi L , Turner SA, Zhao F, Butler G. Gene editing in clinical isolates of *Candida parapsilosis* using CRISPR/Cas9. Sci Rep. 2017;7(1):8051. doi:10.1038/s41598-017-08500-1 28808289 PMC5556056

[jkac301-B72] Lu Z , Lin Z. The origin and evolution of a distinct mechanism of transcription initiation in yeasts. Genome Res. 2021;31(1):51–63. doi:10.1101/gr.264325.120 33219055 PMC7849388

[jkac301-B73] Luesch H , Wu TYH, Ren P, Gray NS, Schultz PG, et al A genome-wide overexpression screen in yeast for small-molecule target identification. Chem Biol. 2005;12(1):55–63. doi:10.1016/j.chembiol.2004.10.015 15664515

[jkac301-B74] MacAlpine J , Daniel-Ivad M, Liu Z, Yano J, Revie NM, et al A small molecule produced by *Lactobacillus* species blocks *Candida albicans* filamentation by inhibiting a *DYRK1*-family kinase. Nat Commun. 2021;12(1):6151. doi:10.1038/s41467-021-26390-w 34686660 PMC8536679

[jkac301-B75] Manoharlal R , Gaur NA, Panwar SL, Morschhäuser J, Prasad R. Transcriptional activation and increased mRNA stability contribute to overexpression of *CDR1* in azole-resistant *Candida albicans*. Antimicrob Agents Chemother. 2008;52(4):1481–1492. doi:10.1128/AAC.01106-07 18268086 PMC2292549

[jkac301-B76] Maroc L , Fairhead C. A new inducible CRISPR-Cas9 system useful for genome editing and study of double-strand break repair in *Candida glabrata*. Yeast. 2019;36(12):723–731. doi:10.1002/yea.3440 31423617

[jkac301-B77] Marr KA , Lyons CN, Ha K, Rustad TR, White TC. Inducible azole resistance associated with a heterogeneous phenotype in *Candida albicans*. Antimicrob Agents Chemother. 2001;45(1):52–59. doi:10.1128/AAC.45.1.52-59.2001 11120944 PMC90239

[jkac301-B78] Marr KA , Lyons CN, Rustad TR, Bowden RA, White TC. Rapid, transient fluconazole resistance in *Candida albicans* is associated with increased mRNA levels of *CDR*. Antimicrob Agents Chemother. 1998;42(10):2584–2589. doi:10.1128/AAC.42.10.2584 9756759 PMC105901

[jkac301-B79] McMillan J , Lu Z, Rodriguez JS, Ahn T-H, Lin Z. YeasTSS: an integrative web database of yeast transcription start sites. Database. 2019;2019:baz048. doi:10.1093/database/baz048 PMC6484093 31032841

[jkac301-B80] Min K , Biermann A, Hogan DA, Konopka JB. Genetic analysis of *NDT80* family transcription factors in *Candida albicans* using new CRISPR-Cas9 approaches. mSphere. 2018;3:e00545-18. doi:10.1128/mSphere.00545-18 10.1128/mSphere.00545-18 PMC6249646 30463924

[jkac301-B81] Min K , Ichikawa Y, Woolford CA, Mitchell AP. *Candida albicans* gene deletion with a transient CRISPR-Cas9 system. mSphere. 2016;1:e00130-16. doi:10.1128/mSphere.00130-16 10.1128/mSphere.00130-16 PMC4911798 27340698

[jkac301-B82] Momen-Roknabadi A , Oikonomou P, Zegans M, Tavazoie S. An inducible CRISPR interference library for genetic interrogation of *Saccharomyces cerevisiae* biology. Commun Biol. 2020;3(1):723. doi:10.1038/s42003-020-01452-9 33247197 PMC7695836

[jkac301-B83] Morio F , Lombardi L, Butler G. The CRISPR toolbox in medical mycology: state of the art and perspectives. PLoS Pathog. 2020;16(1):e1008201. doi:10.1371/journal.ppat.1008201 PMC6964833 31945142

[jkac301-B169] Motaung TE , Ells R, Pohl CH, Albertyn J, Tsilo TJ. Genome-wide functional analysis in *Candida albicans*. Virulence. 2017;8:1563–1579. doi:10.1080/21505594.2017.1292198 28277904 PMC5810496

[jkac301-B170] Mount HO , Revie NM, Todd RT, Anstett K, Collins C, et al Global analysis of genetic circuitry and adaptive mechanisms enabling resistance to the azole antifungal drugs. PLoS Genet. 2018;14:e1007319. doi:10.1371/journal.pgen.1007319 29702647 PMC5922528

[jkac301-B84] Mózsik L , Hoekzema M, de Kok NAW, Bovenberg RAL, Nygård Y, et al CRISPR-based transcriptional activation tool for silent genes in filamentous fungi. Sci Rep. 2021;11(1):1118. doi:10.1038/s41598-020-80864-3 33441979 PMC7806857

[jkac301-B85] Muñoz IV , Sarrocco S, Malfatti L, Baroncelli R, Vannacci G. CRISPR-Cas for fungal genome editing: a new tool for the management of plant diseases. Front Plant Sci. 2019;10:135. doi:10.3389/fpls.2019.00135 30828340 PMC6384228

[jkac301-B86] Ng H , Dean N. Dramatic improvement of CRISPR/Cas9 editing in *Candida albicans* by increased single guide RNA expression. mSphere. 2017;2:e00385-16. doi:10.1128/mSphere.00385-16 10.1128/mSphere.00385-16 PMC5397569 28435892

[jkac301-B87] Nguyen N , Quail MMF, Hernday AD. An efficient, rapid, and recyclable system for CRISPR-mediated genome editing in *Candida albicans*. mSphere. 2017;2:e00149-17. doi:10.1128/mSphereDirect.00149-17 PMC5422035 28497115

[jkac301-B88] Niimi M , Niimi K, Takano Y, Holmes AR, Fischer FJ, et al Regulated overexpression of *CDR1* in *Candida albicans* confers multidrug resistance. J Antimicrob Chemother. 2004;54(6):999–1006. doi:10.1093/jac/dkh456 15486081

[jkac301-B172] Noble SM , French S Kohn LA, Chen V, Johnson AD. Systematic screens of a *Candida albicans* homozygous deletion library decouple morphogenetic switching and pathogenicity. Nat Genet. 2010;42:590–598. doi:10.1038/ng.605 20543849 PMC2893244

[jkac301-B173] Nobile CJ , Fox EP, Nett JE, Sorrells TR, Mitrovich QM, et al A recently evolved transcriptional network controls biofilm development in *Candida albicans*. Cell. 2012;148:126–138. doi:10.1016/j.cell.2011.10.048 22265407 PMC3266547

[jkac301-B89] Nobile CJ , Schneider HA, Nett JE, Sheppard DC, Filler SG, et al Complementary adhesin function in *C. albicans* biofilm formation. Curr Biol. 2008;18(14):1017–1024. doi:10.1016/j.cub.2008.06.034 18635358 PMC2504253

[jkac301-B90] Nødvig CS , Nielsen JB, Kogle ME, Mortensen UH. A CRISPR-Cas9 system for genetic engineering of filamentous fungi. PLoS One. 2015;10(7):e0133085. doi:10.1371/journal.pone.0133085 PMC4503723 26177455

[jkac301-B174] O’Meara TR , Veri AO, Ketela T, Jiang B, Roemer T, et al Global analysis of fungal morphology exposes mechanisms of host cell escape. Nat Commun. 2015;6:6741. 10.1038/ncomms7741 PMC4382923 25824284

[jkac301-B91] Ouedraogo J-P , Tsang A. CRISPR Cas systems for fungal research. Fungal Biol Rev. 2020;34(4):189–201. doi:10.1016/j.fbr.2020.10.002

[jkac301-B92] Park Y-N , Morschhäuser J. Tetracycline-inducible gene expression and gene deletion in *Candida albicans*. Eukaryot Cell. 2005;4(8):1328–1342. doi:10.1128/EC.4.8.1328-1342.2005 16087738 PMC1214539

[jkac301-B93] Payen C , Thompson D. The renaissance of yeasts as microbial factories in the modern age of biomanufacturing. Yeast. 2019;36(12):685–700. doi:10.1002/yea.3439 31423599

[jkac301-B94] Peng D , Tarleton R. EuPaGDT: a web tool tailored to design CRISPR guide RNAs for eukaryotic pathogens. Microb Genom. 2015;1:e000033. doi:10.1099/mgen.0.000033 28348817 10.1099/mgen.0.000033 PMC5320623

[jkac301-B95] Pérez JC . *Candida albicans* dwelling in the mammalian gut. Curr Opin Microbiol. 2019;52:41–46. doi:10.1016/j.mib.2019.04.007 31132744

[jkac301-B96] Perfect JR . The antifungal pipeline: a reality check. Nat Rev Drug Discov. 2017;16(9):603–616. doi:10.1038/nrd.2017.46 28496146 PMC5760994

[jkac301-B97] Peters JM , Silvis MR, Zhao D, Hawkins JS, Gross CA, et al Bacterial CRISPR: accomplishments and prospects. Curr Opin Microbiol. 2015;27:121–126. doi:10.1016/j.mib.2015.08.007 26363124 PMC4659716

[jkac301-B98] Polvi EJ , Veri AO, Liu Z, Hossain S, Hyde S, et al Functional divergence of a global regulatory complex governing fungal filamentation. PLoS Genet. 2019;15(1):e1007901. doi:10.1371/journal.pgen.1007901 PMC6336345 30615616

[jkac301-B99] Prasad R , Banerjee A, Khandelwal NK, Dhamgaye S. The ABCs of *Candida albicans* multidrug transporter Cdr1. Eukaryot Cell. 2015;14(12):1154–1164. doi:10.1128/EC.00137-15 26407965 PMC4664872

[jkac301-B100] Prasad R , De Wergifosse P, Goffeau A, Balzi E. Molecular cloning and characterization of a novel gene of Candida albicans, CDR1, conferring multiple resistance to drugs and antifungals. Curr Genet. 1995;27(4):320–329. doi:10.1007/BF00352101 7614555

[jkac301-B101] Prieto D , Román E, Alonso-Monge R, Pla J. Overexpression of the transcriptional regulator *WOR1* increases susceptibility to bile salts and adhesion to the mouse gut mucosa in *Candida albicans*. Front Cell Infect Microbiol. 2017;7:389. doi:10.3389/fcimb.2017.00389 28955659 PMC5600957

[jkac301-B102] Radzisheuskaya A , Shlyueva D, Müller I, Helin K. Optimizing sgRNA position markedly improves the efficiency of CRISPR/dCas9-mediated transcriptional repression. Nucleic Acids Res. 2016;44(18):e141. doi:10.1093/nar/gkw583 27353328 PMC5062975

[jkac301-B103] Rai LS , van Wijlick L, Chauvel M, d’Enfert C, Legrand M, et al Overexpression approaches to advance understanding of *Candida albicans*. Mol Microbiol. 2021;117(3):589–599. doi:10.1111/mmi.14818 34569668 10.1111/mmi.14818 PMC9298300

[jkac301-B104] Razzaq I , Berg MD, Jiang Y, Genereaux J, Uthayakumar D, et al The SAGA and NuA4 component Tra1 regulates *Candida albicans* drug resistance and pathogenesis. Genetics. 2021;219(2):iyab131. doi:10.1093/genetics/iyab131 PMC8633099 34849885

[jkac301-B171] Roemer T , Jiang B, Davison J, Ketela T, Veillette K, et al Large-scale essential gene identification in *Candida albicans* and applications to antifungal drug discovery. Mol Microbiol. 2003;50:167–181. doi:10.1046/j.1365-2958.2003.03697.x 14507372

[jkac301-B105] Román E , Coman I, Prieto D, Alonso-Monge R, Pla J. Implementation of a CRISPR-based system for gene regulation in Candida albicans. mSphere. 2019;4:e00001-19. doi:10.1128/mSphere.00001-19 PMC6374588 30760608

[jkac301-B106] Román E , Prieto D, Alonso-Monge R, Pla J. New insights of CRISPR technology in human pathogenic fungi. Future Microbiol. 2019;14:1243–1255. doi:10.2217/fmb-2019-0183 31625446

[jkac301-B107] Rosiana S , Zhang L, Kim GH, Revtovich AV, Uthayakumar D, et al Comprehensive genetic analysis of adhesin proteins and their role in virulence of *Candida albicans*. Genetics. 2021;217(2):iyab003. doi:10.1093/genetics/iyab003 PMC8045720 33724419

[jkac301-B108] Roux I , Woodcraft C, Hu J, Wolters R, Gilchrist CLM, et al CRISPR-mediated activation of biosynthetic gene clusters for bioactive molecule discovery in filamentous fungi. ACS Synth Biol. 2020;9(7):1843–1854. doi:10.1021/acssynbio.0c00197 32526136

[jkac301-B109] Ruben S , Garbe E, Mogavero S, Albrecht-Eckardt D, Hellwig D, et al Ahr1 and Tup1 contribute to the transcriptional control of virulence-associated genes in *Candida albicans*. mBio. 2020;11(2):e00206–20. doi:10.1128/mBio.00206-20 32345638 PMC7188989

[jkac301-B175] Ryan O , Shapiro RS, Kurat CF, Mayhew D, Baryshnikova A, et al Global gene deletion analysis exploring yeast filamentous growth. Science 2012;337:1353–1356. doi:10.1126/science.1224339 22984072

[jkac301-B110] Sahni N , Yi S, Daniels KJ, Huang G, Srikantha T, et al Tec1 mediates the pheromone response of the white phenotype of *Candida albicans*: insights into the evolution of new signal transduction pathways. PLoS Biol. 2010;8(5):e1000363. doi:10.1371/journal.pbio.1000363 PMC2864266 20454615

[jkac301-B111] Sanglard D , Coste A, Ferrari S. Antifungal drug resistance mechanisms in fungal pathogens from the perspective of transcriptional gene regulation. FEMS Yeast Res. 2009;9(7):1029–1050. doi:10.1111/j.1567-1364.2009.00578.x 19799636

[jkac301-B112] Sanglard D , Kuchler K, Ischer F, Pagani JL, Monod M, et al Mechanisms of resistance to azole antifungal agents in *Candida albicans* isolates from AIDS patients involve specific multidrug transporters. Antimicrob Agents Chemother. 1995;39(11):2378–2386. doi:10.1128/AAC.39.11.2378 8585712 PMC162951

[jkac301-B113] Sanguinetti M , Posteraro B, Lass-Flörl C. Antifungal drug resistance among *Candida* species: mechanisms and clinical impact. Mycoses. 2015;58(2):2–13. doi:10.1111/myc.12330 10.1111/myc.12330 26033251

[jkac301-B114] Sanjana NE . Genome-scale CRISPR pooled screens. Anal Biochem. 2017;532:95–99. doi:10.1016/j.ab.2016.05.014 27261176 PMC5133192

[jkac301-B115] Sanson KR , Hanna RE, Hegde M, Donovan KF, Strand C, et al Optimized libraries for CRISPR-Cas9 genetic screens with multiple modalities. Nat Commun. 2018;9(1):5416. doi:10.1038/s41467-018-07901-8 30575746 PMC6303322

[jkac301-B116] Santana DJ , O’Meara TR. Forward and reverse genetic dissection of morphogenesis identifies filament-competent *Candida auris* strains. Nat Commun. 2021;12(1):7197. doi:10.1038/s41467-021-27545-5 34893621 PMC8664941

[jkac301-B117] Schmittgen TD , Livak KJ. Analyzing real-time PCR data by the comparative C(T) method. Nat Protoc. 2008;3(6):1101–1108. doi:10.1038/nprot.2008.73 18546601

[jkac301-B118] Schmitz ML , Baeuerle PA. The p65 subunit is responsible for the strong transcription activating potential of NF-kappa B. EMBO J. 1991;10(12):3805–3817. doi:10.1002/j.1460-2075.1991.tb04950.x 1935902 PMC453117

[jkac301-B119] Schubert S , Barker KS, Znaidi S, Schneider S, Dierolf F, et al Regulation of efflux pump expression and drug resistance by the transcription factors Mrr1, Upc2, and Cap1 in *Candida albicans*. Antimicrob Agents Chemother. 2011;55(5):2212–2223. doi:10.1128/AAC.01343-10 21402859 PMC3088179

[jkac301-B120] Schultenkämper K , Brito LF, Wendisch VF. Impact of CRISPR interference on strain development in biotechnology. Biotechnol Appl Biochem. 2020;67(1):7–21. doi:10.1002/bab.1901 32064678

[jkac301-B121] Schuster M , Kahmann R. CRISPR-Cas9 genome editing approaches in filamentous fungi and oomycetes. Fungal Genet Biol. 2019;130:43–53. doi:10.1016/j.fgb.2019.04.016 31048007

[jkac301-B122] Segrelles-Calvo G , de S Araújo GR, Llopis-Pastor E, Carrillo J, Hernández-Hernández M, et al *Candida* spp. Co-infection in COVID-19 patients with severe pneumonia: prevalence study and associated risk factors. Respir Med. 2021;188:106619. doi:10.1016/j.rmed.2021.106619 34555702 PMC8445759

[jkac301-B123] Selmecki A , Forche A, Berman J. Aneuploidy and isochromosome formation in drug-resistant *Candida albicans*. Science. 2006;313(5785):367–370. doi:10.1126/science.1128242 16857942 PMC1717021

[jkac301-B124] Selmecki A , Gerami-Nejad M, Paulson C, Forche A, Berman J. An isochromosome confers drug resistance in vivo by amplification of two genes, ERG11 and TAC1. Mol Microbiol. 2008;68(3):624–641. doi:10.1111/j.1365-2958.2008.06176.x 18363649

[jkac301-B125] Shan L , Dai Z, Wang Q. Advances and opportunities of CRISPR/Cas technology in bioengineering non-conventional yeasts. Front Bioeng Biotechnol. 2021;9:765396. doi:10.3389/fbioe.2021.765396 34708030 PMC8542773

[jkac301-B126] Shapiro RS , Chavez A, Collins JJ. CRISPR-based genomic tools for the manipulation of genetically intractable microorganisms. Nat Rev Microbiol. 2018;16(6):333–339. doi:10.1038/s41579-018-0002-7 29599458

[jkac301-B127] Shapiro RS , Chavez A, Porter CBM, Hamblin M, Kaas CS, et al A CRISPR-Cas9-based gene drive platform for genetic interaction analysis in *Candida albicans*. Nat Microbiol. 2018;3(1):73–82. doi:10.1038/s41564-017-0043-0 29062088 PMC5832965

[jkac301-B128] Sharma J , Rosiana S, Razzaq I, Shapiro RS. Linking cellular morphogenesis with antifungal treatment and susceptibility in Candida pathogens. J Fungi. 2019;5(1):17. doi:10.3390/jof5010017 PMC6463059 30795580

[jkac301-B129] Smith AM , Ammar R, Nislow C, Giaever G. A survey of yeast genomic assays for drug and target discovery. Pharmacol Ther. 2010;127(2):156–164. doi:10.1016/j.pharmthera.2010.04.012 20546776 PMC2923554

[jkac301-B130] Smith JD , Suresh S, Schlecht U, Wu M, Wagih O, et al Quantitative CRISPR interference screens in yeast identify chemical-genetic interactions and new rules for guide RNA design. Genome Biol. 2016;17(1):45. doi:10.1186/s13059-016-0900-9 26956608 PMC4784398

[jkac301-B131] Sopko R , Huang D, Preston N, Chua G, Papp B, et al Mapping pathways and phenotypes by systematic gene overexpression. Mol Cell. 2006;21(3):319–330. doi:10.1016/j.molcel.2005.12.011 16455487

[jkac301-B132] Todd RT , Selmecki A. Expandable and reversible copy number amplification drives rapid adaptation to antifungal drugs. eLife. 2020;9:e58349. doi:10.7554/eLife.58349 32687060 PMC7371428

[jkac301-B133] Todd RT , Wikoff TD, Forche A, Selmecki A. Genome plasticity in *Candida albicans* is driven by long repeat sequences. eLife. 2019;8:e45954. doi:10.7554/eLife.45954 31172944 PMC6591007

[jkac301-B134] Todor H , Silvis MR, Osadnik H, Gross CA. Bacterial CRISPR screens for gene function. Curr Opin Microbiol. 2021;59:102–109. doi:10.1016/j.mib.2020.11.005 33285498 PMC8331264

[jkac301-B135] Uthayakumar D , Sharma J, Wensing L, Shapiro RS. CRISPR-based genetic manipulation of *Candida* species: historical perspectives and current approaches. Front Genome Ed. 2021;2:606281. doi:10.3389/fgeed.2020.606281 34713231 PMC8525362

[jkac301-B136] van Rhijn N , Furukawa T, Zhao C, McCann BL, Bignell E, et al Development of a marker-free mutagenesis system using CRISPR-Cas9 in the pathogenic mould *Aspergillus fumigatus*. Fungal Genet Biol. 2020;145:103479. doi:10.1016/j.fgb.2020.103479 33122116 PMC7768092

[jkac301-B137] Vyas VK , Barrasa MI, Fink GR. A *Candida albicans* CRISPR system permits genetic engineering of essential genes and gene families. Sci Adv. 2015;1(3):e1500248. doi:10.1126/sciadv.1500248 PMC4428347 25977940

[jkac301-B138] Vyas VK , Guy Bushkin G, Bernstein DA, Getz MA, Sewastianik M, et al New CRISPR mutagenesis strategies reveal variation in repair mechanisms among fungi. mSphere. 2018;3:e00154-18. 10.1128/mSphere.00154-18 PMC5917429 29695624

[jkac301-B139] Wang P . Two distinct approaches for CRISPR-Cas9-mediated gene editing in *Cryptococcus neoformans* and related species. mSphere. 2018;3:e00208-18. doi:10.1128/mSphereDirect.00208-18 10.1128/mSphereDirect.00208-18 PMC6001613 29898980

[jkac301-B140] Wang H , La Russa M, Qi LS. CRISPR/Cas9 in genome editing and beyond. Annu Rev Biochem. 2016;85(1):227–264. doi:10.1146/annurev-biochem-060815-014607 27145843 PMC13384723

[jkac301-B141] Wensing L , Shapiro RS. Design and generation of a CRISPR interference system for genetic repression and essential gene analysis in the fungal pathogen *Candida albicans*. Methods Mol Biol. 2022;2377:69–88. doi:10.1007/978-1-0716-1720-5_4 34709611

[jkac301-B142] Wensing L , Sharma J, Uthayakumar D, Proteau Y, Chavez A, et al A CRISPR interference platform for efficient genetic repression in *Candida albicans*. mSphere. 2019;4(1):e00002-19. doi:10.1128/mSphere.00002-19 PMC6374589 30760609

[jkac301-B143] White TC . Increased mRNA levels of *ERG16*, *CDR*, and *MDR1* correlate with increases in azole resistance in *Candida albicans* isolates from a patient infected with human immunodeficiency virus. Antimicrob Agents Chemother. 1997;41(7):1482–1487. doi:10.1128/AAC.41.7.1482 9210670 PMC163944

[jkac301-B144] Wilson FM , Harrison RJ. CRISPR/Cas9 mediated editing of the Quorn fungus *Fusarium venenatum* A3/5 by transient expression of Cas9 and sgRNAs targeting endogenous marker gene PKS12. Fungal Biol Biotechnol. 2021;8(1):15. doi:10.1186/s40694-021-00121-8 34789333 PMC8597179

[jkac301-B145] Xu X , Qi LS. A CRISPR–dCas toolbox for genetic engineering and synthetic biology. J Mol Biol. 2019;431(1):34–47. doi:10.1016/j.jmb.2018.06.037 29958882

[jkac301-B146] Yang F , Teoh F, Tan ASM, Cao Y, Pavelka N, et al Aneuploidy enables cross-adaptation to unrelated drugs. Mol Biol Evol. 2019;36(8):1768–1782. doi:10.1093/molbev/msz104 31028698 PMC6657732

[jkac301-B147] Yang F , Todd RT, Selmecki A, Jiang Y-Y, Cao Y-B, et al The fitness costs and benefits of trisomy of each *Candida albicans* chromosome. Genetics. 2021;218(2):iyab056. doi:10.1093/genetics/iyab056 PMC8225349 33837402

[jkac301-B148] Zhang C , Meng X, Wei X, Lu L. Highly efficient CRISPR mutagenesis by microhomology-mediated end joining in *Aspergillus fumigatus*. Fungal Genet Biol. 2016;86:47–57. doi:10.1016/j.fgb.2015.12.007 26701308

[jkac301-B149] Zhang X-H , Tee LY, Wang X-G, Huang Q-S, Yang S-H. Off-target effects in CRISPR/Cas9-mediated genome engineering. Mol Ther Nucleic Acids. 2015;4:e264. doi:10.1038/mtna.2015.37 26575098 PMC4877446

[jkac301-B150] Zhao X , Oh S-H, Cheng G, Green CB, Nuessen JA, et al *ALS3* And *ALS8* represent a single locus that encodes a *Candida albicans* adhesin; functional comparisons between Als3p and Als1p. Microbiology. 2004;150(7):2415–2428. doi:10.1099/mic.0.26943-0 15256583

[jkac301-B151] Zhao X-Q , Zhang X-Y, Zhang F, Zhang R, Jiang B-J, et al 2018. Metabolic engineering of fungal strains for efficient production of cellulolytic enzymes. In: Fang X, Qu Y, editors. Fungal Cellulolytic Enzymes: Microbial Production and Application. Singapore: Springer. p. 27–41.

[jkac301-B152] Znaidi S , van Wijlick L, Hernández-Cervantes A, Sertour N, Desseyn J-L, et al Systematic gene overexpression in *Candida albicans* identifies a regulator of early adaptation to the mammalian gut. Cell Microbiol. 2018;20(11):e12890. doi:10.1111/cmi.12890 29998470 PMC6220992

[jkac301-B153] Zoppo M , Luca MD, Villarreal SN, Poma N, Barrasa MI, et al A CRISPR/Cas9-based strategy to simultaneously inactivate the entire *ALS* gene family in *Candida orthopsilosis*. Future Microbiol. 2019;14(16):1383–1396. doi:10.2217/fmb-2019-0168 31659913

